# Examining predictors of cocaine withdrawal syndrome at the end of detoxification treatment in women with cocaine use disorder

**DOI:** 10.1016/j.jpsychires.2023.11.043

**Published:** 2024-01

**Authors:** Bernardo Aguzzoli Heberle, Bruno Kluwe-Schiavon, Carla Bicca, Leonardo Melo Rothmann, Rodrigo Grassi-Oliveira, Thiago Wendt Viola

**Affiliations:** a Department of Neuroscience, College of Medicine, University of Kentucky, KY, USA; b Department of Psychiatry and Behavioral Sciences, The University of Texas Health Science Center at Houston, Houston, TX, USA; c School of Medicine, Brain Institute of Rio Grande do Sul, Developmental Cognitive Neuroscience Lab, Pontifical Catholic University of Rio Grande do Sul (PUCRS), Porto Alegre, RS, Brazil; d Translational Neuropsychiatry Unit, Department of Clinical Medicine, Aarhus University, Aarhus, Denmark

**Keywords:** Cocaine use disorder, Machine learning, Withdrawal, Crack cocaine, Childhood maltreatment

## Abstract

Detoxification is frequently recommended as a treatment for moderate to severe Cocaine Use Disorder (CUD). However, the response to detoxification varies among patients, and previous studies have focused mostly on patterns of drug use behavior to test associations with treatment outcomes, overlooking the potential impact of psychosocial factors, other clinical variables, and individual life experiences. In this study we comprehensively examined several variables aiming to find the most relevant predictors to classify patients with severe versus non-severe cocaine withdrawal symptoms at the end of detoxification. Methods: Data from 284 women with CUD who enrolled in a 3-week detoxification program was used in this longitudinal study. Psychosocial, clinical, and drug use behavior characteristics were evaluated, generating a dataset with 256 potential predictors. We tested six different machine learning classification algorithms. Results: The best classification algorithm achieved an average accuracy and ROC-AUC of approximately 70%. The 16 features selected as best predictors were the severity of psychiatric, family, and social problems and the level of exposure to childhood maltreatment. Features associated with drug-use behavior included days consuming drugs and having craving symptoms in the last month before treatment, number of previous drug/alcohol-related treatments, and a composite score of addiction severity. The level of cocaine withdrawal syndrome at the beginning of detoxification was also a key feature for classification. A network analysis revealed the pattern of association between predictors. Conclusion: These variables can be assessed in real-world clinical settings, potentially helping clinicians to identify individuals with severe cocaine withdrawal that is likely to be sustained over the course of detoxification.

## Highlights

• Machine learning is a promising method to identify patients at high risk of severe withdrawal.
• Almost seventy percent of accuracy was achieved by using machine learning.
• The top features included drug use behavior, childhood maltreatment, and psychiatric problems.

## 1 Introduction

Cocaine Use Disorder (CUD) is a major public health issue associated with poor outcomes for both the patients and society (United Nations Office on Drugs and Crime, 2022). Inpatient detoxification treatment is often recommended as a treatment for moderate to severe Cocaine Use Disorder (CUD), especially for those individuals who regularly consume cocaine and are at high risk of severe intoxication, craving, overdose, or withdrawal. The assessment for this indication also considers co-occurring substance use disorders, mental health and emotional needs, medical and physical requirements, as well as social and environmental factors affecting patients (Schwartz et al., 2022). Inpatient treatment is also considered when individuals experience significant addiction-related issues necessitating a protected environment, when maintaining abstinence is challenging, or when minimizing exposure to cocaine-related triggers is essential.

However, the response to inpatient detoxification treatment varies among patients, as some individuals face greater challenges in managing the withdrawal syndrome than others (CSAT, 2006). Therefore, an important clinical outcome measure for CUD detoxification response is the management of the severity of cocaine withdrawal syndrome, including symptoms of craving, anxiety, irritability, mood changes, physiological manifestations (e.g. fatigue, restlessness, sleepiness, appetite alterations, tremors, muscle aches, nerve pain) and even suicidal ideation (Kampman, 2009, 2010). The identification of patients with severe cocaine withdrawal syndrome during early treatment stages would be helpful in clinical practice since these patients have the lowest odds of successfully maintaining abstinence and other positive long-term outcomes (Ahmadi et al., 2008; Viola et al., 2014). For instance, the results of the Cocaine Selective Severity Assessment (CSSA) for withdrawal syndrome, obtained at the start of inpatient/outpatient treatment, have been shown to predict later treatment outcomes in CUD, such as continuous abstinence (Kampman et al., 2002), and treatment droupout (Ahmadi et al., 2009). In addition, people with CUD with elevated CSSA scores exhibited a higher prevalence of psychiatric comorbidities and reported more family history of problematic substance use, both of which are important indicators of difficult-to-treat patients (Ahmadi et al., 2008). Thus, routine screening and assessment of the most relevant factors related to the CSSA score could help identify patients who would most benefit from more extensive and personalized interventions.

Some studies have analyzed variables related to cocaine withdrawal syndrome and abstinence during outpatient or detoxification treatment. For instance, Ahmadi and collaborators (2009) demonstrated that the baseline treatment variables most consistently predicting patients who achieved four weeks of abstinence from cocaine, compared to those who did not, were the initial urine drug screen results, the initial CSSA scores, and initial self-reported cocaine use in the past 30 days. In contrast, cocaine craving, alcohol craving, and alcohol withdrawal symptoms did not play significant roles. Viola and collaborators (2020) found that patients with CUD and frequent recent cannabis use reported higher severity of cocaine withdrawal after three weeks of treatment, while recent alcohol or tobacco use did not show an association with increased CSSA scores. In addition to substance use variables, some studies have shown that psychosocial experiences can also influence the severity of cocaine withdrawal syndrome. For example, Francke and collaborators (2013) presented findings indicating that women with CUD and a history of childhood neglect exposure exhibited a significantly lower reduction in the severity of cocaine withdrawal symptoms during three weeks of treatment compared to those with CUD but without a history of childhood neglect.

While the aforementioned studies have examined a subset of factors individually, in the current study, we comprehensively analyzed more than 250 variables collected from women with CUD who enrolled in a 3-week inpatient treatment program for detoxification. Taking advantage of capturing complex nonlinear relationships, multiple variables, and robustness to outliers (Bzdok et al., 2018), machine learning (ML) was used to develop a predictive model for a treatment outcome estimated by cocaine withdrawal syndrome severity. Precisely, we examined psychosocial, medical, legal, psychiatric, and family characteristics, as well as history/patterns of substance use, and traumatic experiences variables, and tested six different classification algorithms to predict severe versus non-severe cocaine withdrawal syndrome at the end of the detoxification treatment. We utilized a SHAP (SHapley Additive exPlanations) and a regularized partial correlation network analysis to assess the contribution of each feature to the model predictions and explore the relationships between these features (Stewart, 2019). Furthermore, this study utilized data from women with CUD, a population that has historically been underrepresented in substance use research.

## 2 Materials and methods

### 2.1 Source of data and participants

This was a longitudinal study performed during 3 weeks of a detoxification program. We adapted the TRIPOD guidelines for reporting predictive models and the checklist of items is found as Supplementary Table 1 (Heus et al., 2019). The data was collected from women with CUD who underwent voluntary hospitalization in an inpatient psychiatric unit for drug detoxification managed by the Brazilian Universal Healthcare System in the city of Porto Alegre. The treatment program was in an all-female unit and took place over 21 days of detoxification and drug rehabilitation, and included standard psychoeducation, support groups, moderate physical activity, balanced diet (2200 Kcal/day), nursing care, psychological/psychiatric treatment, and medical treatment. The psychological treatment focused on support and psychoeducation related to substance use disorders including relapse prevention training, coping techniques, motivational interviewing, monitoring of symptoms, and strategies for treatment adherence after discharge. The psychopharmacological treatment consisted primarily of neuroleptics for the management of psychomotor agitation. Additional medications such as antidepressants, mood stabilizers, and other neuroleptics could be prescribed depending on the case.

The participants met the following criteria: (1) age between 18 and 45 years old; (2) diagnosis of cocaine use disorder according to the SCID-I; and (3) absence of any neurological disorder, severe medical condition, or primary psychotic disorder. The data collection process occurred over seven years between 2012 and 2019. The administered instruments were the CSSA (Kampman et al., 1998; Kluwe-Schiavon et al., 2015) (14, 15) to assess cocaine withdrawal symptoms, the Addiction Severity Index Version 6 (ASI-6) (Cacciola et al., 2011; Kessler et al., 2012) to assess drug use characteristics, addiction severity, and problems in other areas of psychosocial functioning (e.g., medical, legal, psychiatric, family and social problems), and the Childhood Trauma Questionnaire (CTQ) (Bernstein et al., 1994; Grassi-Oliveira et al., 2006) to assess childhood maltreatment exposure. The CSSA was administered twice. Once in the first week of treatment and again in the third - and last - week of treatment, before discharge. Also, participants underwent the Structured Clinical Interview (SCID-I) based on the Diagnostic and Statistical Manual of Mental Disorders (DSM-V) criteria to confirm their CUD diagnosis.

The CSSA is a clinician-administered instrument with 18 items that measure early cocaine abstinence withdrawal-related symptoms like depression, fatigue, carbohydrate craving, cocaine craving, anhedonia, anxiety, irritability, bradycardia, sleep disturbance, suicidality, and inability to concentrate. Each item was scored from 0 to 7 with the maximum total score being 112. The ASI-6 is a semi-structured interview that assesses the severity of drug addiction. The main fields of interest are “medical problems”, “legal issues”, “occupational”, “psychiatric symptoms”, “family problems”, “history/patterns of substance use”, “social support”, and “traumatic experiences”. This instrument generates variables (quantitative and qualitative, such as yes/no responses) and a total score for each field of interest (possible score range: 25–80). The substances covered by the ASI-6 are alcohol, cannabis, crack, cocaine, opioids, inhalants, sedatives, stimulants, hallucinogens, and tobacco. The CTQ is a self-answered 5-point Likert-type questionnaire with 28 items that evaluate the severity of childhood maltreatment exposure. The results include a total score (possible score range: 25–125) and sub-scores for five types of maltreatment (emotional abuse, physical abuse, sexual abuse, emotional neglect, and physical neglect), each ranging between 5 and 25 points. All the instruments were fulfilled by trained research staff.

### 2.2 Data pre-processing and outcome

**Fig. 1:**
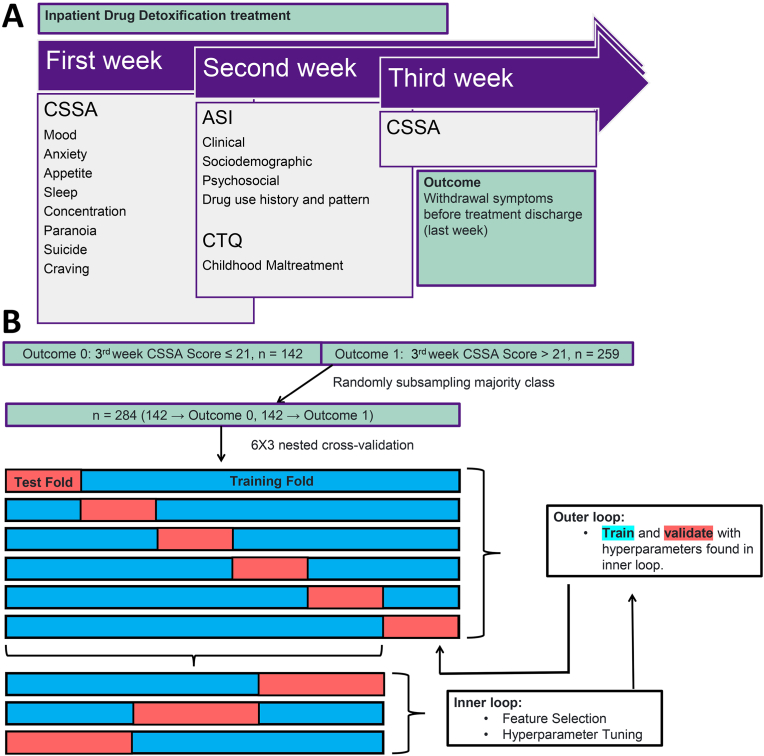
Study Design Note. A - Data collection process. B - Machine Learning approach. After randomly undersampling, machine learning algorithms were generated with data from participants with severe (n = 142) and non-severe (n = 142) withdrawal syndrome at the end of treatment, based on the cut-off of 21 points in the CSSA. Nested cross-validation was performed with six rounds in the outer loop and three rounds in the inner loop. ASI-6 - Addiction Severity Index, 6 edition; CSSA - Cocaine Selective Severity Assessment; CTQ - Childhood Trauma Questionnaire.

Our initial dataset contained 525 female CUD patients, with 402 who completed the detoxification treatment (77%). Since our main goal was to evaluate withdrawal severity at the end of detoxification, we only included patients who completed the treatment program in the analyses. The main outcome was severe versus non-severe cocaine withdrawal symptoms at the end of detoxification (Fig. 1). Our cutoff point for this outcome was based on previous study where CSSA score was dichotomized along the entire range of scores and each dichotomous variable was entered into a separate logistic regression model (Kampman et al., 2002). Therefore, Kampman et al. (2002) found that patients with a CSSA score above 21 were 12 times more likely to fail to achieve three weeks of continuous cocaine abstinence. This finding was validated by urine toxicology screen results and self-reports.

Based on the evidence from their study, we split our initial sample containing data from 402 patients who completed the detoxification treatment between severe withdrawal (outcome 1, n = 259, CSSA score > 21) and non-severe withdrawal (outcome 0, n = 142, CSSA score ≤ 21). Because this split resulted in imbalanced sample sizes between outcomes, which could bias ML results toward the outcome with higher frequency, we applied an under-sampling procedure. We used the function RandomUnderSampler from the imbalanced-learn Python package to randomly under-sample the majority class, resulting in a dataset with 284 participants and an equal number of patients assigned to severe (n = 142) and non-severe withdrawal symptoms (n = 142).

The initial dataset contained 292 variables without missing values. No imputation procedure was performed. Non-numerical binary and ordinal categorical variables were converted into numerical values. If the categorical variable was binary, values were converted into 1s and 0s. If the categorical variable was ordinal, each level was converted into a number corresponding to the ordinal scale of the variable (i.e., a variable with ordinal categorical values such as low, medium, high would be converted to 0, 1, 2 respectively). Nominal categorical variables and variables with statistical dispersion equal to zero were excluded from the dataset. The CSSA score from the last week of treatment was also excluded since it was used to create the outcome being predicted. Any variables with no or low variance were removed. A variable was considered to have low variance if a single value accounted for 95% or more of the feature entries. Thus, the final dataset was composed of 256 potential predictors. The variables included in the initial dataset are presented in Supplementary Table 2, and the final dataset is presented in Supplementary Table 3.

### 2.3 Feature selection, nested cross-validation, and hyperparameter tuning

Because of the high number of potential predictive features, a comprehensive feature selection approach was performed with the Python package scikit-learn using the function SelectFromModel. This function allows the user to specify a classification algorithm and uses the feature importance values calculated by that algorithm to return the “n” best features. We chose the random forest algorithm as our classifier for feature selection.

To do so, we used nested cross-validation, which is a method used to evaluate the performance of a ML model by using two layers of cross-validation. In this method, an outer cross-validation loop is used to split the data into training (80%) and test sets (20%), and an inner loop is used to tune the hyperparameters of the model using the training set (Fig. 1). The performance of the model is then evaluated on the test set. In our approach, the outer loop was used to split the data into six folds (five training folds and one testing fold). The inner loop then puts the five outer loop training folds together and splits them into three folds (two training and one testing). Feature selection and hyperparameter tuning were performed within the inner loop cross-validation. The inner loop trains the model using different numbers of features and hyperparameter configurations. It then selects the best one based on the prediction performance for the inner loop test set. Finally, the selected model is re-fitted on the entire outer loop training set and the performance is evaluated on the outer loop test set to obtain an estimate of the model's generalization performance. This process was repeated for each round of cross-validation in the outer loop (six times in total). Within each inner loop of nested cross-validation, we applied the SelectFromModel function returning either six, eight, ten, or twelve features. Hyperparameter tuning in the inner loop of nested cross-validation was performed using each of the four possible numbers of selected features (six, eight, ten, or twelve). The hyperparameter tunning search space used for each classification algorithm is reported in the supplementary materials (Supplementary Tables 4 and 5). The advantage of nested cross-validation is that it provides a more accurate - less overly optimistic - estimate of the model's performance on unseen data.

Following features selection, six ML classification algorithms were used in our analysis (Logistic Regression, Random Forest, Naïve Bayes, Light Gradient Boosting Machine, Support Vector Machine, and K-Nearest Neighbors), implemented in Python version 3.10.5 using the scikit-learn package version 1.1.1 (Pedregosa et al., 2015). Including multiple algorithms from different families is a common practice in machine learning research, since they have different underlying assumptions, learning mechanisms, and strengths, which makes their inclusion valuable for a comprehensive analysis.

### 2.4 Model evaluation and interpretation

The metrics used for model evaluation were accuracy, precision, recall, F1-score, and ROC-AUC. The average and standard deviation of performance metrics for the model predictions on the outer loop test set were reported using nested cross-validation. We used the SHAP package (Lundberg et al., 2020) to interpret the best model and a regularized partial correlation network was used to explore a possible relationship structure between our predictors (Heeren et al., 2020). Shapley values are defined as the average marginal contribution of a feature value across all included features in the model, enabling the visualization of features ordered according to its importance. The SHAP values reported come from the models for each of the six outer loop cross-validation rounds applied to their respective test datasets.

Information regarding the strength and shape of the associations between features has been suggested as an additional tool for understanding ML models (Molnar et al., 2022). Correlation-based network analysis has been used to aid the understanding of ML results (Toubiana et al., 2019). Partial network analysis estimates partial associations between two features while controlling for the associations between all other features in the network. To ensure the stability of the expected influence, a person-dropping bootstrap procedure was performed. Moreover, only for the purposes of the network analysis, we applied a nonparanormal transformation via the R package “huge” (Zhao et al., 2012). The least absolute shrinkage and selection operator (LASSO) graphical algorithm was used to perform the network with the R package “qgraph”. The regularized partial network analysis was performed with features included in at least two of the outer loop cross validation rounds for the best classification algorithm.

## 3 Results

Sociodemographic characteristics of the groups are presented in Table 1, and group comparisons of all the remaining variables are presented in Supplementary Table 3. Regarding CSSA scores (Table 1), we also performed a repeated measures ANOVA including time (beginning and discharge) and group (severe versus non-severe withdrawal) as factors, detecting significant effects for time (F = 135; p < 0.0001), group (F = 248; p < 0.0001) and an interaction between time and group (F = 36; p < 0.0001). This shows that patients with higher CSSA scores at the beginning of detoxification do not achieve as great a reduction in symptoms after 3 weeks of treatment as those with lower CSSA scores.

**Table 1:** Sociodemographic characteristics of the two groups.

*Variables*	Non-severe withdrawal	Severe withdrawal	**Statistics**	***p*-value**
Age, mean (SD)	31.91 (8.94)	33.57 (9.28)	t = 1.4	0.161
Monthly income, mean (SD)^a^	375.4 (618.1)	763.2 (1848.1)	t = 2.3	0.018
*Ethnicity (%)*			χ^2^ = 1.1	0.774
Black	59 (50.0)	56 (52.3)		
White	47 (39.8)	44 (41.1)		
Mixed	11 (9.3)	6 (5.6)		
Indigenous/Native	1 (0.8)	1 (0.9)		
*Marital status (%)*			χ^2^ = 10.5	0.061
Married	8 (6.6)	16 (14.4)		
Stable union	34 (27.9)	29 (26.1)		
Widow	7 (5.7)	6 (5.4)		
Divorced	7 (5.7)	0 (0.0)		
Separated	31 (25.4)	24 (21.6)		
Single	35 (28.7)	36 (32.4)		
*Education level (%)*			χ^2^ = 3	0.552
None	62 (52.5)	58 (59.2)		
Elementary school	42 (35.6)	30 (30.6)		
High school	13 (11.0)	9 (9.2)		
Graduation/Bachelor	1 (0.8)	0 (0.0)		
Master or more	0 (0.0)	1 (1.0)		
Alcohol regular use, mean (SD)^b^	4.64 (7.84)	3.73 (6.85)	t = 1.04	0.296
Nicotine regular use, mean (SD)^b^	15.03 (10.5)	14.38 (10.07)	t = 0.5	0.597
Cannabis regular use, mean (SD)^b^	2.44 (4)	3.19 (4.59)	t = 1.4	0.146
Cocaine regular use, mean (SD)^b^	6.16 (5.28)	6.91 (5.07)	t = 1.2	0.226
CSSA score first week, mean (SD)	32.1 (18.1)	48.3 (17.9)	t = 7.5	<0.001
CSSA score third week, mean (SD)^c^	12.4 (5.6)	42 (16.2)	t = 20.4	<0.001

Note: CSSA - Cocaine Selective Severity Assessment; χ^2^ - chi-square test; t - independent samples *t*-test; SD - standard deviation; ^a^ - The Brazilian real was converted for the purpose of comparison with US dollars using the purchasing power parity function, as per The World Bank's data. ^b^ – Years of regular use (a minimum of three days of use per week for 12 months); ^c^ - Variable used to estimate the outcome of the study.

### 3.1 Prediction models and performance measures

The six classifier algorithms were moderately able to distinguish patients with severe versus non-severe withdrawal syndrome at the week of treatment discharge, with accuracy and ROC-AUC greater than 60%. The average and standard deviation of performance metrics for the model predictions on the outer loop test set are reported in Table 2. The best classification algorithm was the logistic regression, achieving an accuracy of 69.7%, ROC-AUC of 69.6%, precision of 68.2%, recall of 73.8%, and F1-score of 70.8%. Supplementary Table 4 contains the hyperparameter search space used for each classification algorithm, while Supplementary Table 5 contains the hyperparameters used in the six logistic regression models that yielded the best results when tested in the outer loop of the nested cross-validation.

**Table 2:** 6x3 nested cross-validation results.

	KNN	SVM	Log Reg	Light GBM	NB	RF
Accuracy	62.0%(5.2%)	69.0%(4.6%)	69.7%(3.8%)	69.4%(5.6%)	65.1%(5.4%)	66.9%(2.8%)
Precision	65.3%(7.3%)	70.2%(3.2%)	68.2%(4.9%)	68.9%(6.9%)	65.7%(6.3%)	67.3%(6.1%)
Recall	52.4%(9.7%)	65.4%(9.8%)	73.8%(3.5%)	71.1%(11.8%)	60.9%(14.8%)	67.4%(9.8%)
F1	57.5%(7.4%)	67.4%(6.4%)	70.8%(3.9%)	69.4%(6.9%)	62.6%(9.8%)	66.7%(4.3%)
ROC-AUC	61.5%(5.0%)	68.8%(4.7%)	69.6%(3.9%)	69.4%(5.8%)	64.9%(5.8%)	66.7%(3.2%)

Note: KNN – K-Nearest Neighbors. SVM - Support Vector Machine. Log Reg - Logistic Regression. Light GBM - Light Gradient Boosting Machine. NB - Naïve Bayes. RF - Random Forest. Number in parenthesis is the the average standard deviation from the six outer folds of the nested cross validation.

The 16 features included in the best ML algorithm (logistic regression) with at least one round of outer loop cross-validation were the following: 1) ASI-6 drug-related problems score; 2) CTQ total score; 3) CSSA score of the first week of treatment; 4) ASI-6 psychiatric difficulties score; 5) ASI-6 drug-related craving symptoms in the last month before treatment; 6) ASI-6 number of previous drug/alcohol related treatments; 7) ASI-6 family/social-related problems score; 8) CTQ emotional abuse subscore; 9) CTQ emotional neglect subscore; 10) ASI-6 days consuming drugs in the last month before treatment; 11) CTQ sexual abuse subscore; 12) ASI-6 ratio between age and years of regular use of nicotine; 13) ASI-6 days consuming crack in the lkast month before treatment; 14) ASI-6 days consuming cocaine or crack in the last month before treatment; 15) ASI-6 days in controlled environment in the last month before treatment; and 16) ASI-6 ratio between age and years of regular use of cannabis. Detailed information about each of the 16 features is found in Table 3. A correlation matrix between all 16 features is presented in Supplementary Fig. 1.

**Table 3:** Detailed information about each of the 16 features in the best model.

	Variable	Nature	Description
1.	ASI-6 drug-related problems score	Continous	Composite score of features related to polysubstance use patterns and problems managing drug use behavior during the last 30 days prior to treatment enrollment
2.	CTQ total score	Continous	Composite score of the 5 types of childhood maltreatment exposure (physical abuse, emotional abuse, sexual abuse, physical neglect, emotional neglect
3.	CSSA score of the first week of treatment	Continous	Severity of cocaine withdrawal syndrome in the beginning of detoxification
4.	ASI-6 psychiatric difficulties score	Continous	Composite score of features related to depressive, anxiety, psychotic, cognitive, impulsivity, aggressive, and suicide ideation symptoms during the last 30 days prior to treatment. It also accounts for the need and motivation for trauma-related treatment and psychological suffering
5.	ASI-6 drug-related craving symptoms in the last month before treatment	Dichotomic	Self-report acknowledgment of craving symptoms for any substance in the last 30 days prior to detoxification treatment
6.	ASI-6 number of previous drug/alcohol related treatments	Continous	Sum of lifetime previous drug/alcohol related treatments, including inpatient and outpatient treatments
7.	ASI-6 family/social-related problems score	Continous	Composite score of features related to problems with partners, friends, and family members during the last 30 days prior to detoxification
8.	CTQ emotional abuse subscore	Continous	Severity of exposure to emotional abuse experiences during childhood
9.	CTQ emotional neglect subscore	Continous	Severity of exposure to emotional neglect experiences during childhood
10.	ASI-6 days consuming drugs in the last month before treatment	Continous	Days with any substance use during the last month prior to detoxification
11.	CTQ sexual abuse subscore	Continous	Severity of exposure to sexual abuse experiences during childhood
12.	ASI-6 ratio between age and years of regular use of nicotine	Continous	Number of years smoking nicotine at least 3 days per week, divided by chronological age
13.	ASI-6 days consuming crack in the last month before treatment	Continous	Number of days consuming crack prior to treatment
14.	ASI-6 days consuming cocaine or crack in the last month before treatment	Continous	Number of days consuming snorted cocaine prior to treatment
15.	ASI-6 days in controlled environment in the last month before treatment	Continous	Number of days in controlled environments (hospitalization, prison or police station, sheltered pension, therapeutic community) prior to detoxification
16.	ASI-6 ratio between age and years of regular use of cannabis	Continous	Number of years smoking cannabis at least 3 days per week, divided by chronological age

Note: ASI-6 - Addiction Severity Index, 6 edition; CSSA - Cocaine Selective Severity Assessment; CTQ - Childhood Trauma Questionnaire.

### 3.2 Model interpretation

**Fig. 2:**
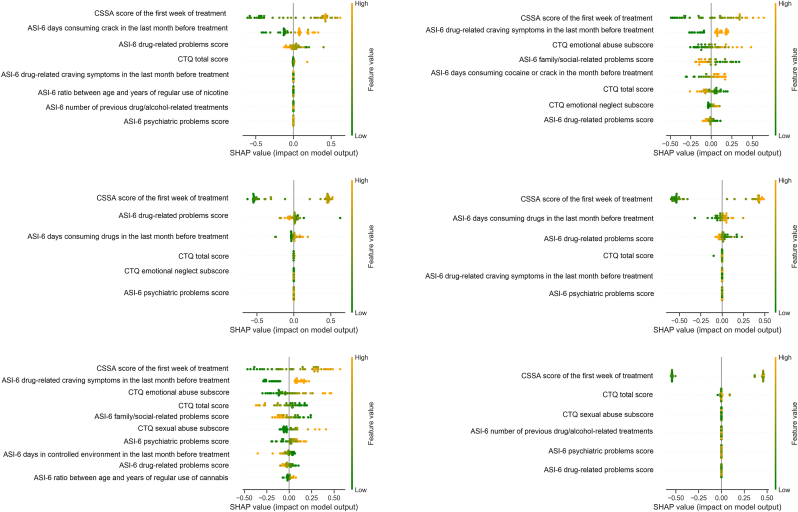
SHAP Summary Plot for logistic regression model predictions in each outer loop of nested cross-validation. Note: An orange dot on the right side of the graph indicates that a high feature value corresponds to a contribution towards a prediction of severe withdrawal. Note. SHAP Summary Plot demonstrating the contribution of each feature to the model prediction. Dots with an orange color indicate a higher feature value, whereas dots with a green color indicate a lower feature value. The more to the right of the graph the greater the contribution of that feature towards a prediction of a severe withdrawal outcome and vice-versa. ASI-6 - Addiction Severity Index, 6 edition; CSSA - Cocaine Selective Severity Assessment; CTQ - Childhood Trauma Questionnaire; Psychiatric problems means difficulties.

**Fig. 3:**
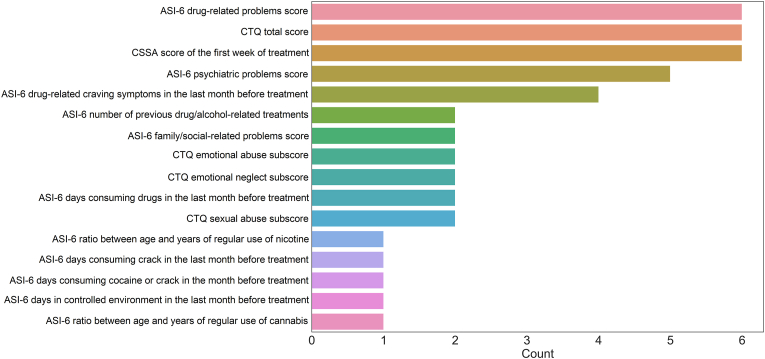
Number of times features were included in the outer loop of nested cross-validation for the logistic regression classification algorithm. Note: ASI-6 - Addiction Severity Index, 6 edition; CSSA - Cocaine Selective Severity Assessment; CTQ - Childhood Trauma Questionnaire; Psychiatric problems means difficulties.

In Fig. 2 we have a plot of SHAP values from the best logistic regression model for each of the six outer loop cross-validation rounds. The SHAP values reported are respective to the test dataset in each round of outer loop cross validation. These SHAP values help us interpret the ML model by calculating the contribution of each feature to the prediction. The features are displayed in order of importance from top to bottom. The CSSA score of the first week of treatment appeared as the top feature in all 6 rounds of cross-validation. The features “ASI-6 days consuming drugs in the last month before treatment” and “ASI-6 drug-related craving symptoms in the last month before treatment” had the second highest importance in two out of six rounds. The features “CTQ total score” and “ASI-6 drug-related problems score” also occupied the second highest importance in one round. In Fig. 3, we provide the number of times features were included in each outer loop of cross-validation. ASI-6 drug-related problems score, CTQ total score, and CSSA score of the first week of treatment were the only features included in all six rounds of cross-validation. The ASI-6 psychiatric difficulties score was included in five out of six rounds, while the binary feature - drug-related craving symptoms in the last month before treatment – was included in four rounds.

**Fig. 4:**
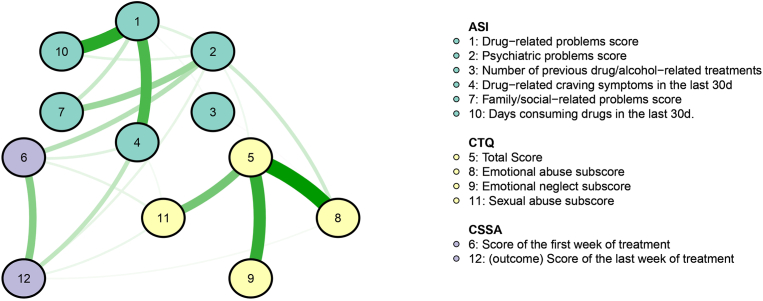
Regularized partial network analysis of the top features Note. The thickness of an edge reflects the magnitude of the association. Green full lines represent positive regularized partial correlations. Yellow nodes represent variables related to childhood maltreatment experiences. Green nodes represent variables related to addiction severity, history of treatments and drug use behavior, psychiatric problems (difficulties) score and family/social-related problems score. Purple nodes represent variables related to cocaine withdrawal. The hyperparameter γ was set in 0.5, favoring a simpler model containing fewer edges. ASI-6 - Addiction Severity Index, 6 edition; CSSA - Cocaine Selective Severity Assessment; CTQ - Childhood Trauma Questionnaire.

We performed regularized partial network analysis using features included in at least two rounds of outer loop cross-validation (Fig. 4). The network analysis revealed that the outcome variable is not only positively correlated with the CSSA score of the first week of treatment - as expected - but also with drug-related craving symptoms in the last month before treatment, ASI-6 psychiatric difficulties score, and CTQ sexual and emotional abuse sub-scores. In addition, the CSSA score of the first week of treatment had a strong correlation with ASI-6 psychiatric difficulties score. The network also highlighted a cluster of CTQ variables in the lower right showing high correlation between childhood maltreatment experiences. It is worth mentioning that the CTQ emotional abuse score positively correlated with the ASI-6 psychiatric difficulties score, connecting the childhood maltreatment-related variables with the addiction severity cluster (ASI-6 variables). The upper left cluster shows the correlation pattern between ASI-6 variables, for instance, the strong correlation between days consuming drugs in the last month before treatment and drug-related problems score. A strong correlation between psychiatric difficulties score and family/social-related problems score was also evident.

## 4 Discussion

In this study we aimed to predict which female patients will have a CSSA score higher than 21 points at treatment discharge, and to identify the most relevant features of widely used instruments in addiction research (CSSA, ASI-6, and CTQ). Out of 256 variables, 16 features were selected to generate the models for best performing this classification. These features included the severity of psychiatric, family, and social problems and the level of exposure to childhood maltreatment. Features associated with drug-use behavior were also included, such as days consuming drugs and having craving symptoms in the last month before treatment enrollment, lifetime number of previous drug/alcohol-related treatments, and a composite score associated with the severity of addiction. The severity of cocaine withdrawal syndrome at the beginning of detoxification was also a key feature for classification. The algorithm with the best performance achieved an accuracy close to 70%.

A large body of literature supports that the presence and severity of withdrawal symptoms are key predictors of future substance use patterns (Allen et al., 2008), biopsychosocial problems related to substance use disorder (Hasin et al., 2000), and relapse following detoxification discharge. Although some patients may show a complete relief from withdrawal symptoms after one or two weeks of treatment, psychostimulants like cocaine can lead to a prolonged withdrawal syndrome lasting up to a month (Alsheikh, 2021; Fox et al., 2007). As shown in Table 1, the CSSA scores decreased from the first week (non-severe withdrawal: 32.1 points/severe withdrawal: 48.3) to the third week (non-severe withdrawal: 12.4 points/severe withdrawal: 42) of treatment in both groups. Consistent with prior studies involving a similar sample (Francke et al., 2013; Rovaris et al., 2015; Viola et al., 2020), however, we demonstrated that even after three weeks of detoxification some patients may continue to report high CSSA scores. In this sense, it is important to note that our study focused on patients with a high level of CUD severity. These patients reported consuming cocaine for over 19 days out of the last 30 days before treatment and had an average of at least 6 years of regular cocaine consumption (see Supplementary Table 3). This underscores the necessity for research to identify factors contributing to severe and sustained cocaine withdrawal syndrome in these individuals. We observed that the ASI-6 drug-related problems score, the CTQ total score, and the CSSA score of the first week of treatment were the features included in all six rounds of cross-validation of the best ML algorithm. We consider these features as having the highest significant potential for future clinical applications.

The drug-related problems score is estimated by several variables from the ASI-6 (see Supplementary Table 1), including how many days the patient consumed substances such as nicotine, alcohol, sedatives, cannabis, and cocaine, in the last month before treatment enrollment. In addition, the composite score also incorporates variables related to the amount of money spent on drug use, how worried the patient feels about problems due to substance use, and how important the current treatment is for the patient. Therefore, the drug-related problems score shows a comprehensive view of the severity of the substance use disorder, highlighting the pattern of polysubstance use and the subjective motivation for drug abstinence. A higher drug-related problems score was associated with the severe withdrawal outcome at the end of detoxification, suggesting that a broad assessment of the recent drug use behavior - not only focused on the drug of preference - is necessary when managing cocaine withdrawal syndrome.

The CTQ total score is estimated by the sum of all five types of childhood maltreatment experiences assessed by the instrument, including distinct forms of abuse and neglect. In particular, the sub-scores of childhood emotional abuse/neglect and childhood sexual abuse were also present among the 16 included features in the best model. Several studies have shown that childhood abuse and neglect are major risk factors for the development of substance use disorders, including CUD (Santo et al., 2021; Werner et al., 2016; Zhang et al., 2020). A history of childhood maltreatment has also been shown to be associated with earlier experimentation of drugs (Andersen and Teicher, 2009; Tractenberg et al., 2012), higher depressive and cocaine craving/withdrawal symptoms (Francke, I.D.a. et al., 2013), and more severe cognitive deficits associated with CUD (Viola et al., 2013). These findings support the merits of the current comprehensive approach, by showing that factors that were salient in other analyses, were also detected as relevant features in our analysis. Importantly, the network analysis revealed that childhood maltreatment experiences were highly correlated, as expected due to their prevalent co-occurrence (de Azeredo et al., 2019).

The ASI-6 psychiatric difficulties score, which was also an important feature appearing in 5 out of 6 rounds of cross-validation loops, is generated by several features including depressive, anxiety, psychotic, cognitive, impulsivity, aggressive, and suicide ideation symptoms during the last 30 days prior to detoxification, depicting a general dimension of psychiatric morbidity that is not directly related to substance use behavior. It also accounts for the need and motivation for trauma-related treatment and psychological suffering. The network analysis revealed that childhood maltreatment variables were associated with the ASI-6 psychiatric difficulties score, highlighting that early interventions tailored to address childhood abuse and neglect may provide a window of opportunity to prevent the influence of these early adversities on the risk of developing CUD later in life (Andersen and Teicher, 2009). In addition, psychiatric difficulties score was strongly associated with the severity of cocaine withdrawal symptoms at the beginning of treatment. This highlights that the severity of cocaine withdrawal syndrome is affected by multiple factors of distinct natures, and not only by the pattern of cocaine use *per se*, suggesting that integrated interventions that target substance use and co-occurring mental health conditions of anxiety, depression, and/or PTSD during detoxification treatment are needed (Schwartz et al., 2022). Furthermore, since childhood maltreatment is a risk factor not only for substance use disorders but also for mood, anxiety, and trauma-related disorders (Norman et al., 2012), our findings of the association between CTQ and psychiatric difficulties suggest that PTSD or anxiety/depression symptoms resulting from childhood maltreatment could increase withdrawal severity. These considerations also apply to emotional abuse as a form of childhood maltreatment, in addition to experiences of sexual abuse. As observed in our network analysis, there was an association between emotional abuse score and ASI psychiatric difficulties score. This finding underscores the importance of assessing emotional abuse as a traumatic experience in individuals with substance use disorders (Evren et al., 2011), which is often overlooked by standard screening methods for comorbid conditions such as PTSD.

Additionally, we observed a group per time interaction, indicating that high CSSA scores at baseline may not resolve as quickly over time as low CSSA scores. This finding was supported by the ML analysis, which identified baseline CSSA scores as a highly important feature for group classification. This suggests that levels of cocaine withdrawal syndrome at the beginning of detoxification treatment are indeed associated with cocaine withdrawal at discharge. Thus, emphasizing the importance of multiple assessments of withdrawal symptoms throughout the period of early abstinence. This also supports the findings of Ahmadi et al. (2009), showing that the CSSA score in the beginning of treatment is a relevant predictor of treatment outcome throughout the first month of abstinence.

We had some limitations that should be considered. First, our sample consisted only of women. While studies on CUD have historically underrepresented women, it is important to note that the current findings should not be extrapolated to men without further investigation. Gender plays a significant role in shaping differences in clinical outcomes among individuals with CUD (Becker et al., 2017). While CUD prevalence is higher in men, data suggests that women exhibit higher CUD severity and experience more challenges in areas related to childcare, involvement in criminal activities, work-related issues, social support, and more childhood maltreatment exposure (Sanvicente-Vieira et al., 2019). Additionally, women with CUD demonstrate greater sensitivity to the impact of interpersonal problems on craving and relapse compared to men (Becker et al., 2016; Hartwell and Ray, 2013). Although our study findings cannot be generalized to men, they underscore the importance of gender-specific treatment interventions in detoxification units. This is particularly important given the influence of psychosocial factors that may interact with gender-specific considerations and impact the outcomes of detoxification treatment. Second, this study was conducted with patients enrolled in an inpatient detoxification treatment unit, and these findings cannot be generalized to other CUD treatment approaches such as outpatient services and harm-reduction strategies. Third, the CSSA is a widely used instrument in addiction research, but it has been used dimensionally. Kampman et al. (2002) showed that a score greater than 21 points in the CSSA is an indicator of much lower likelihood to maintain cocaine abstinence, therefore, we applied this cut-off to dichotomize our withdrawal outcome measure. However, we did not assess relapse and withdrawal syndrome after treatment discharge, which are important outcomes to be tested in the future. Fourth, although our sample size was larger than previous attempts at developing ML algorithms in substance use disorder research (Fu et al., 2023; Mak et al., 2019), it was still relatively small, and overfitting could have been an issue. However, we used nested cross-validation to develop our models, which is a robust preventative measure against overfitting.

In conclusion, using a large dataset with several characteristics of women receiving detoxification treatment for CUD in a naturalistic setting, it was observed that both psychosocial variables and clinical features relate to drug use behavior, when assessed at treatment baseline, can help to predict cocaine withdrawal syndrome before treatment discharge. In terms of implications, the 16 variables included in the model with the best performance, can be easily assessed in real-world clinical settings and could help clinicians assess and manage cocaine withdrawal symptoms during early abstinence among women.

## Authors agreement

The authors were solely responsible for the entire work, including the study conception and design, material preparation, data collection and analysis, and writing of the manuscript. All authors have approved the final article. The authors declare there is no conflict of interest.

## Funding

This study was supported by the Bill and Melinda Gates Foundation - Grand Challenges Explorations - Brazil grant for Data Science approaches to improve Maternal, Women and Child Health in Brazil: NV027961; NIDA and Fogarty Foundation: R01DA044859; Secretaria Nacional de Cuidados e Prevenção às Drogas: 822647/2015; FAPERGS: 21/2551-0000125-9; and by CNPq: 466802/2014-5.

## Data availability

Available upon request.

## Code availability

Scripts used for the machine learning analyses included in this paper can be found here: https://github.com/bernardo-heberle/dcnl_ml_predicting_detox_outcomes.

## CRediT authorship contribution statement

**Bernardo Aguzzoli Heberle:** Data curation, Formal analysis, Methodology, Writing – original draft. **Bruno Kluwe-Schiavon:** Data curation, Formal analysis. **Carla Bicca:** Conceptualization, Methodology. **Leonardo Melo Rothmann:** Data curation, Methodology. **Rodrigo Grassi-Oliveira:** Conceptualization, Funding acquisition, Writing – review & editing. **Thiago Wendt Viola:** Conceptualization, Funding acquisition, Methodology, Supervision, Writing – review & editing.

## Declaration of competing interest

The authors declare there is no conflict of interest. The study was approved by the institutional review boards of included institutions, and it was performed in accordance with the ethical standards as laid down in the 1964 Declaration of Helsinki.

## Appendix A Supplementary data

Multimedia component 1 Supplementary Fig. 1. Correlation matrix with heat map between the 16 included features in the logistic regression best model Note: Pearson's correlation matrix. Green color represents positive correlations, while red color represents negative correlations. ASI-6 - Addiction Severity Index, 6 edition; CSSA - Cocaine Selective Severity Assessment; CTQ - Childhood Trauma Questionnaire. Multimedia component 1
