# Supplementary figures and images for: Examining predictors of cocaine withdrawal syndrome at the end of detoxification treatment in women with cocaine use disorder
Source: J Psychiatr Res. 2024 Jan;169:247–56. doi: 10.1016/j.jpsychires.2023.11.043 (PMC10805009; doi:10.1016/j.jpsychires.2023.11.043)

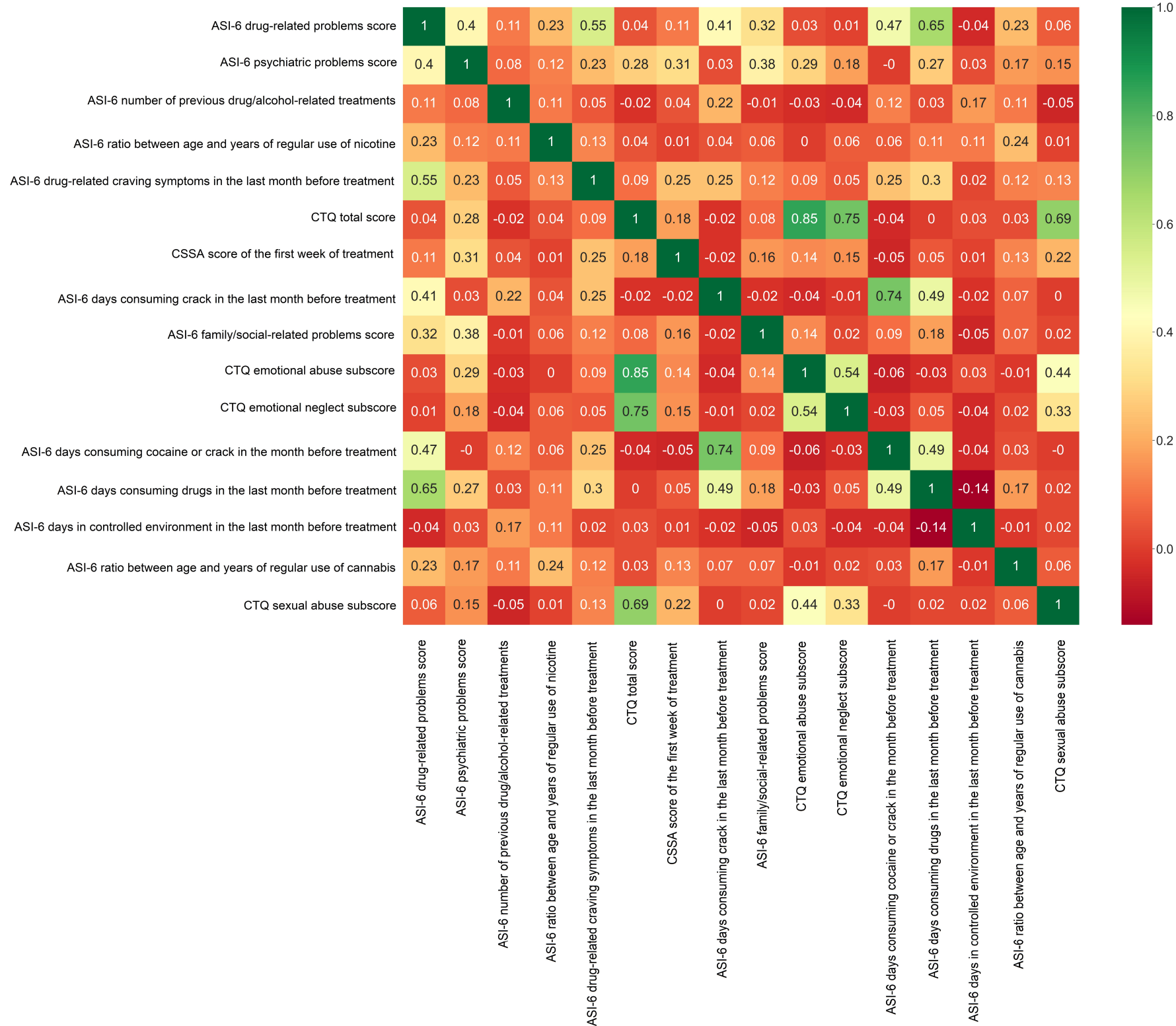

Supplement: Multimedia component 1 — Supplementary Fig. 1. Correlation matrix with heat map between the 16 included features in the logistic regression best model Note: Pearson's correlation matrix. Green color represents positive correlations, while red color represents negative correlations. ASI-6 - Addiction Severity Index, 6 edition; CSSA - Cocaine Selective Severity Assessment; CTQ - Childhood Trauma Questionnaire. [file mmc1.pdf]
