# Supplementary material for: Examining predictors of cocaine withdrawal syndrome at the end of detoxification treatment in women with cocaine use disorder
Source: J Psychiatr Res. 2024 Jan;169:247–56. doi: 10.1016/j.jpsychires.2023.11.043 (PMC10805009; doi:10.1016/j.jpsychires.2023.11.043)
Supplement: Multimedia component 3 [file mmc3.docx]

| **Instrument** | **Original variable name** | **Variable name in the dataset** | **Description** | **ASI Score** |
| --- | --- | --- | --- | --- |
| ASI | D01 | D_AlcDrugsNumberTreatments | How many different times have you been treated for your alcohol or drug use? |  |
| ASI | D02 | D_AlcDrugsDetox | How many of those treatments were just for detox? |  |
| ASI | D04A | D_AlcDrugsOutpatient_6Monhts | In the last 6 months, have you participated in an outpatient program or medical appointment to treat alcohol or drug-related problems? |  |
| ASI | D04B | D_AlcDrugsOutpatient_30Days | In the last 30 days, have you participated in an outpatient program or medical appointment to treat alcohol or drug-related problems? |  |
| ASI | D05B | D_AlcDrugsPrescribedMed_30Days | In the last 6 months, have you taken prescribed medication to treat your alcohol or drug use? |  |
| ASI | D06B | D_AA_30Days | In the last 6 months, have you participated in self-help meetings (eg AA, NA)? |  |
| ASI | D07 | D_AALongestPeriod_Months | In months, what is the longest continuous period of time that you have attended self-help meetings, at least 2 days/week? |  |
| ASI | D08 | D_AlcRegularUse_Yrs | How many years in your life did you drink alcohol regularly, 3 or more days/week? |  |
| ASI | D09 | D_AlcRegularAbuse_Yrs | How many years in your life have you had at least (5-male, 4-female) drinks1 a day on a regular basis, 3 or more days a week? |  |
| ASI | D10 | D_Alc50DaysMore | Have you had at least (5 – male, 4 – female) drinks per day on 50 or more days in your life? |  |
| ASI | D12 | D_AlcFrequencyASI_week | In the past 6 months, during the month when you were drinking the most, how often did you drink? |  |
| ASI | D13 | D_AlcUse_30Days | In the past 30 days, how many days did you drink any type of alcoholic beverage? | ASI_Alcohol |
| ASI | D14 | D_AlcAbstinence_Dys | When did you last drink? | ASI_Alcohol |
| ASI | D15 | D_AlcAbuse_30Days | In the last 30 days, how many days did you have at least (5 for men, 4 for women) drinks in one day? | ASI_Alcohol |
| ASI | D16 | D_AlcMoneySpent | In the last 30 days, how much money did you spend on alcohol for yourself? | ASI_Alcohol |
| ASI | D17 | D_AlcAbstinenceSymptom | In the last 30 days, did you have any withdrawal symptoms shortly after you cut down or stopped drinking? | ASI_Alcohol |
| ASI | D18 | D_AlcUncontrolledSymtom | In the last 30 days, did you have any difficulty controlling, cutting down or stopping your drinking or did you spend a large part of the day drinking? | ASI_Alcohol |
| ASI | D19 | D_AlcMedPsychSymtom | In the last 30 days, because of your drinking, have you had any medical or psychological problems; or had problems at work (school) or at home, had arguments; or had trouble with the law? | ASI_Alcohol |
| ASI | D20 | D_AlcEverCrave | In the last 30 days, have you been bothered by cravings or intense cravings to drink? | ASI_Alcohol |
| ASI | D21 | D_AlcProblems_30Days | In the last 30 days, how many days did you have these or any other difficulties due to alcohol use? | ASI_Alcohol |
| ASI | D22 | D_AlcTroubleBother_scl | In the last 30 days, how worried or bothered have you been about these alcohol problems? | ASI_Alcohol |
| ASI | D23 | D_AlcTreatImportance_scl | At this time, how important is treatment (current or additional) for your alcohol use to you? | ASI_Alcohol |
| ASI | D25 | D_CannabisUse_Life | Have you ever tried cannabis? |  |
| ASI | D25B | D_Cannabis_Yrs | For how many years of your life did you use cannabis 3 or more days a week? |  |
| ASI | D25B* | D_CannabisRegAbuseRatio | D25B divided by Age |  |
| ASI | D25C | D_Cannabis50DaysMore | Have you used cannabis on 50 or more days in your life? |  |
| ASI | D25D | D_Cannabis_30Days | In the last 30 days, how many days did you use cannabis? | ASI_Drugs |
| ASI | D25E | D_CannabisAsTreatment | In the past 30 days, have you used cannabis as a treatment? | ASI_Drugs |
| ASI | D26 | D_SedativeUse_Life | Have you ever tried sedatives? |  |
| ASI | D26B | D_Sedative_Yrs | For how many years of your life did you use sedatives 3 or more days a week? |  |
| ASI | D26B* | D_SedativesRegAbuseRatio | D25B divided by Age |  |
| ASI | D26C | D_Sedative50DaysMore | Have you used sedatives on 50 or more days in your life? |  |
| ASI | D26D | D_Sedative_30Days | In the last 30 days, how many days did you use sedatives? | ASI_Drugs |
| ASI | D26E | D_SedativeAsTreatment | In the past 30 days, have you used sedatives as a treatment? | ASI_Drugs |
| ASI | D2728 | D_CrackCocaineUse_Life | Have you ever tried crack or cocaine? |  |
| ASI | D2728B* | D_CrackCocaine_Yrs | For how many years of your life did you use crack or cocaine 3 or more days a week? |  |
| ASI | D2728D | D_CrackCocaine_30Days | In the last 30 days, how many days did you use crack or cocaine? |  |
| ASI | D27 | D_CocaineUse_Life | Have you ever tried cocaine? |  |
| ASI | D27B | D_Cocaine_Yrs | For how many years of your life did you use cocaine 3 or more days a week? |  |
| ASI | D27B* | D_CocaineRegAbuseRatio | D27B divided by Age |  |
| ASI | D27C | D_Cocaine50DaysMore | Have you used cocaine on 50 or more days in your life? |  |
| ASI | D27D | D_Cocaine_30Days | In the last 30 days, how many days did you use cocaine? | ASI_Drugs |
| ASI | D27E | D_CocaineAsTreatment | In the past 30 days, have you used cocaine as a treatment? | ASI_Drugs |
| ASI | D28 | D_CrackUse_Life | Have you ever tried crack? |  |
| ASI | D28B | D_Crack_Yrs | For how many years of your life did you use crack 3 or more days a week? |  |
| ASI | D28B* | D_CrackRegAbuseRatio | D28B divided by Age |  |
| ASI | D28C | D_Crack50DaysMore | Have you used crack on 50 or more days in your life? |  |
| ASI | D28D | D_Crack_30Days | In the last 30 days, how many days did you use crack? | ASI_Drugs |
| ASI | D28E | D_CrackAsTreatment | In the past 30 days, have you used crack as a treatment? | ASI_Drugs |
| ASI | D29 | D_StimulantsUse_Life | Have you ever tried stimulants? |  |
| ASI | D29B | D_Stimulants_Yrs | For how many years of your life did you use stimulants 3 or more days a week? |  |
| ASI | D29C | D_Stimulants50DaysMore | Have you used stimulants on 50 or more days in your life? |  |
| ASI | D29D | D_Stimulants_30Days | In the last 30 days, how many days did you use stimulants? | ASI_Drugs |
| ASI | D29E | D_StimulantsAsTreatment | In the past 30 days, have you used stimulants as a treatment? | ASI_Drugs |
| ASI | D30 | D_HallucinUse_Life | Have you ever tried hallucinogens? |  |
| ASI | D30B | D_Hallucin_Yrs | For how many years of your life did you use hallucinogens 3 or more days a week? |  |
| ASI | D30C | D_Hallucin50DaysMore | Have you used hallucinogens on 50 or more days in your life? |  |
| ASI | D30D | D_Hallucin_30Days | In the last 30 days, how many days did you use hallucinogens? | ASI_Drugs |
| ASI | D30E | D_HallucinAsTreatment | In the past 30 days, have you used hallucinogens as a treatment? | ASI_Drugs |
| ASI | D31 | D_HeroinUse_Life | Have you ever tried heroin? |  |
| ASI | D31D | D_Heroin_30Days | In the last 30 days, how many days did you use heroin? | ASI_Drugs |
| ASI | D31E | D_HeroinAsTreatment | In the past 30 days, have you used heroin as a treatment? | ASI_Drugs |
| ASI | D32 | D_OpioidUse_Life | Have you ever tried opioids? |  |
| ASI | D32B | D_Opioid_Yrs | For how many years of your life did you use opioids 3 or more days a week? |  |
| ASI | D32C | D_Opioid50DaysMore | Have you used opioids on 50 or more days in your life? |  |
| ASI | D32D | D_Opioid_30Days | In the last 30 days, how many days did you use opioids? | ASI_Drugs |
| ASI | D32E | D_OpioidAsTreatment | In the past 30 days, have you used opioids as a treatment? | ASI_Drugs |
| ASI | D33 | D_InhalantUse_Life | Have you ever tried inhalants? |  |
| ASI | D33B | D_Inhalant_Yrs | For how many years of your life did you use inhalants 3 or more days a week? |  |
| ASI | D33C | D_Inhalant50DaysMore | Have you used inhalants on 50 or more days in your life? |  |
| ASI | D33D | D_Inhalant_30Days | In the last 30 days, how many days did you use inhalants? | ASI_Drugs |
| ASI | D33E | D_InhalantAsTreatment | In the past 30 days, have you used inhalants as a treatment? | ASI_Drugs |
| ASI | D33 | D_NicotineUse_Life | Have you ever tried nicotine/tobacco? |  |
| ASI | D33A* | D_NicotineUseRatio | Age minus D33B, divided by Age |  |
| ASI | D33B | D_Nicotine_Yrs | For how many years of your life did you use nicotine/tobacco 3 or more days a week? |  |
| ASI | D33C | D_Nicotine50DaysMore | Have you used nicotine/tobacco on 50 or more days in your life? |  |
| ASI | D33D | D_Nicotine_30Days | In the last 30 days, how many days did you use nicotine/tobacco? | ASI_Drugs |
| ASI | D33E | D_NicotineAsTreatment | In the past 30 days, have you used nicotine/tobacco as a treatment? | ASI_Drugs |
| ASI | D33G | D_NicotineCraving | From 1 to 10, how much do you crave for nicotine/tobacco right now? |  |
| ASI | D38 | D_DrugFrequencyASI_week | In the past 6 months, during the month when you were using the most illegal or street drugs (and/or abusing prescription medication), how often did you use any drugs? |  |
| ASI | D39 | D_DrugUse_30Days | In the last 30 days, on how many days did you use any type of drug or abuse prescription medication? | ASI_Drugs |
| ASI | D40 | D_DrugAbstinence_Dys | How many days has it been since you last used any type of drug or abused prescription medication? | ASI_Drugs |
| ASI | D41 | D_DrugsSpent | In the last 30 days, how much money did you spend on drugs? | ASI_Drugs |
| ASI | D42 | D_DrugAbstinenceSymptom | In the last 30 days, did you have any withdrawal symptoms shortly after tapering off or stopping any drug? | ASI_Drugs |
| ASI | D43 | D_DrugUncontrolledSymtom | In the last 30 days, did you have any problems controlling, tapering, or stopping drugs, or did you spend much of your day using, high, recovering, or just trying to get drugs? | ASI_Drugs |
| ASI | D44 | D_DrugMedPsychSymtom | In the last 30 days, because of your drug use – you had a medical or psychological problem; or had problems at work (school) or at home, got into arguments; or had trouble with the law? | ASI_Drugs |
| ASI | D45 | D_DrugEverCrave | In the last 30 days, have you been bothered by cravings or cravings to use? | ASI_Drugs |
| ASI | D46 | D_DrugProblems_30Days | In the last 30 days, how many days did you have these or any other difficulties due to drug use? | ASI_Drugs |
| ASI | D47 | D_DrugTroubleBother_scl | In the last 30 days, how worried or bothered have you been about these drug problems? | ASI_Drugs |
| ASI | D48 | D_DrugTreatImportance_scl | In the last 30 days, how important is treatment (current or additional) for your drug use to you? | ASI_Drugs |
| ASI | D57 | D_NicotineDailyUse_Yrs | How many years in your life have you smoked cigarettes (or otherwise used tobacco) on a daily basis? |  |
| ASI | D59 | D_GamblingProblems | In your life, have you ever had financial difficulties because of gambling? |  |
| ASI | D60 | D_GamblingProblems_30Days | In the last 30 days, how many days did you participate in any form of gambling, such as bingo, lottery, horse racing, animal games, cockfights, casinos, or illegal gambling of any kind? |  |
| ASI | E19 | E_PaidWork_30Days | In the last 30 days, how many paid days did you work? | ASI_Employement |
| ASI | E20 | E_GrossIncome_PerMonth_Adjusted | In the last 30 days, how much money did you earn (gross income)? | ASI_Employement |
| ASI | E20* | E_EstimatedTotalGrossIncome_Yrs_PPPadj | In the last 30 days, how much money did you earn (gross income) adjusted by the Purchasing power parity? |  |
| ASI | E21 | E_EmployProbs_30Days | In the last 30 days, how many days did you have any work-related problems? | ASI_Employement |
| ASI | E23 | E_EmployImportance_scl | At this time, how important is receiving any kind of guidance (such as advice, training or education) to help you prepare for or find a job, or deal with professional problems? | ASI_Employement |
| ASI | E24 | E_FinancedHousingAssistance | Do you live in government-funded housing or receive housing assistance? |  |
| ASI | E25A | E_MoneyPension_30Days_Adjusted | In the last 30 days, how much money did you receive from pension, social security, R$ unemployment insurance? |  |
| ASI | E26A | E_MoneyWelfare_30Days_Adjusted | In the last 30 days, how much money did you receive from In the past 30 days, how much money did you receive from public assistance? |  |
| ASI | E27A | E_AdditionalMoneyWelfare_30Days_Adjusted | In the last 30 days, how much money did you receive from other assistance? |  |
| ASI | E28A | E_MoneyAlimony_30Days_Adjusted | In the past 30 days, how much money did you receive in support or child support? |  |
| ASI | E29A | E_MoneyIllegalInformal_30Days | In the last 30 days, how much money did you receive from illegal activities? | ASI_Employement |
| ASI | E30A | E_MoneyOtherSource_30Days_Adjusted | In the last 30 days, how much money did you receive from some other source, borrow/receive family money or unexpected income (inheritance, taxes, lottery, etc.)? |  |
| ASI | E31* | E_EmploymentMainSource | Dummy coded for sources of financial support: Employment |  |
| ASI | E31* | E_RetirementMainSource | Dummy coded for sources of financial support: Retirement |  |
| ASI | E31* | E_HealthPensionMainSource | Dummy coded for sources of financial support: Health pension |  |
| ASI | E31* | E_UnemploymentInsurMainSource | Dummy coded for sources of financial support: Unemployment Insurance |  |
| ASI | E31* | E_PublicWelfareMainSource | Dummy coded for sources of financial support: Public welfare |  |
| ASI | E31* | E_AlimonyMainSource | Dummy coded for sources of financial support: Alimony |  |
| ASI | E31* | E_FriendsFamilyMainSource | Dummy coded for sources of financial support: Family and Friends |  |
| ASI | E31* | L_IlegalMainSource | Dummy coded for sources of financial support: Illegal |  |
| ASI | E31* | E_InstitutionalizedMainSource | Dummy coded for sources of financial support: Institutionalized |  |
| ASI | E32 | E_Bankruptcy | Have you ever declared bankruptcy? |  |
| ASI | E33 | E_PersonalDebt | Have you ever defaulted on a loan to the government or private institution? |  |
| ASI | E34 | E_CurrentPersonalDebt | Are you more than a month late on your payments for something? |  |
| ASI | E36 | E_EnoughtMoneyLiving | Do you have enough income to pay for necessities such as housing, food and clothing for yourself and your dependents? |  |
| ASI | E* | E_CurrentStudying_FullTime | Dummy coded for currently participating in a technical training or educational program: Yes, full time |  |
| ASI | E5* | E_CurrentStudying_No | Dummy coded for currently participating in a technical training or educational program: No |  |
| ASI | E6 | E_DriverLicense | Do you have a valid driver's license? |  |
| ASI | E7 | E_OwnVehicle | Do you use or have a car or motorcycle? |  |
| ASI | E8 | E_LaborStudyingDifficultVehicle | At this moment, is it difficult to go to work/school, or look for work because of the means of transport? |  |
| ASI | E9 | E_WriteRead | Do you read/write (Portuguese) well enough to fill out a job application? |  |
| ASI | F1 | F_RomanticSexualRelationship | Have you had a romantic or sexual relationship with a partner during the past month? |  |
| ASI | F10 | F_JudicianlRemoval | Do you currently have a restraining order against someone? |  |
| ASI | F11 | F_PhysicalAgressionCloseFriends | In the past 30 days, did any situations with your partner, adult relatives, or close friends result in you pushing/hitting or throwing things? | ASI_FamilySocialProblem |
| ASI | F12 | F_SomeoneElseToRely | In addition to your partner, other adult relatives, and close friends, is there anyone you can turn to if you really need help? |  |
| ASI | F14 | F_RelationshipTroubBother_scl | In the last 30 days, how worried or bothered have you been about any problems with your relationships with adults? | ASI_FamilySocialProblem |
| ASI | F15 | F_RelationshipImportance_scl | At this time, how important is it to you to receive help, counseling or treatment (current or additional) for your adult relationship problems? | ASI_FamilySocialProblem |
| ASI | F16 | F_DifficExpressingEmotions | Do you find it difficult to talk about your feelings or problems even with close people (including relatives)? |  |
| ASI | F17 | F_SocialDisconfort | Do you feel nervous or uncomfortable around other people? |  |
| ASI | F18 | F_RelationshipImportance | Is it important to you to have close/intimate relationships with people? |  |
| ASI | F19 | F_AttendedReliousGroup | In the last 30 days, did you attend mass or religious activities organized by your church/congregation? |  |
| ASI | F2 | F_CloseFriends | How many close/true friends do you have? |  |
| ASI | F20 | F_VoluntaryWork | In the last 30 days, did you do any volunteer work? |  |
| ASI | F21 | F_DifficSpendingFreeTime | In the last 30 days, you often felt upset or upset difficulty enjoying your free time? |  |
| ASI | F23 | T_PhysicallyAssaultedSomeoneKnown | Have you ever been physically assaulted/abused by someone you knew? |  |
| ASI | F26 | T_Raped | Have you ever been sexually assaulted/abused by someone? |  |
| ASI | F29 | T_ViolentCrimeBeaten | Have you ever been the victim of a violent crime such as being beaten or assaulted? |  |
| ASI | F3A | F_TimeTogheterPartner | In the last 30 days, spent time (in person) with partner? | ASI_FamilySocialSupport |
| ASI | F3B | F_TimeTogheterFamily | In the last 30 days, spent time (in person) with family? | ASI_FamilySocialSupport |
| ASI | F3C | F_TimeTogheterFriends | In the last 30 days, spent time (in person) with friends? | ASI_FamilySocialSupport |
| ASI | F32 | T_DeathRisk | Have you ever been in any other life-threatening situations? |  |
| ASI | F35 | T_WitnessExtremeViolence | Have you ever been in a situation where you saw someone being killed, beaten/assaulted or seriously injured? |  |
| ASI | F38 | T_TraumaTroubBother_scl | In the past 30 days, how worried or troubled have you been by feelings, thoughts, or other reactions related to these events? | ASI_Psychiatric |
| ASI | F39 | T_TraumaImportance_scl | At this time, how important is it to you to receive help, advice or treatment (current or additional) for any feelings, thoughts or other reactions related to these events? | ASI_Psychiatric |
| ASI | F4A | F_AnyContactPartner | In the last 30 days, had any contact, such as, letters, phone calls or e-mail (other) with partner? | ASI_FamilySocialSupport |
| ASI | F4B | F_AnyContactFamily | In the last 30 days, had any contact, such as, letters, phone calls or e-mail (other) with family? | ASI_FamilySocialSupport |
| ASI | F4C | F_AnyContactFriends | In the last 30 days, had any contact, such as, letters, phone calls or e-mail (other) with friends? | ASI_FamilySocialSupport |
| ASI | F42 | F_OpenCustodyProceedings | Is there any custody case opened by the mother/father or any other relative? |  |
| ASI | F44 | F_ChildrenLivingTogether_30Days | In the past 30 days, how many children (under 18) lived with you at least some of the time? |  |
| ASI | F45 | F_OtherChildLivingTogether_30Days | In the past 30 days, has any other child (stepchild/grandchild/nephew, etc.), under 18, lived with you for at least some time? |  |
| ASI | F46 | F_ChildSeriousProbLivingTogether_30Days | How many of the children (who lived with you) have serious health, behavioral or learning problem(s) that require professional care, treatment or specialized care? | ASI_FamilyChild |
| ASI | F47 | F_ChildSeriousProbNeedForAssistance_scl | At this time, how much additional services are needed to address these issues? | ASI_FamilyChild |
| ASI | F48 | F_ProblemLivingWithChild_scl | In the past 30 days, have you had problems getting along with these children (< 18) who have lived with you for at least some time? | ASI_FamilyChild |
| ASI | F49 | F_ChildcareAssistance_scl | At this point, how important is counseling (eg parenting classes) to you to help you get along better with these children (< 18) who lived with you? | ASI_FamilyChild |
| ASI | F5A | F_ShareFeelingsPartner | In the last 30 days, did you told your partner about your feelings or problems? | ASI_FamilySocialSupport |
| ASI | F5B | F_ShareFeelingsFamily | In the last 30 days, did you told your family about your feelings or problems? | ASI_FamilySocialSupport |
| ASI | F5C | F_ShareFeelingsFriends | In the last 30 days, did you told your friends about your feelings or problems? | ASI_FamilySocialSupport |
| ASI | F50 | F_ChildcareAssistance | Do you currently need more help with childcare in order to participate in drug treatment, work/study or look for work? | ASI_FamilyChild |
| ASI | F51 | F_EverCustodyProcess | Have you ever been investigated or under supervision by the Guardian Council or another child protection program? |  |
| ASI | F52 | F_ChildrenEverProtected | Has your child ever been removed from home by the Tutelary Council or another program? |  |
| ASI | F53 | F_SuspendedRightsChildren | Has your parental power (patrio power) ever been suspended? |  |
| ASI | F54 | F_UnderCustodyProcess | Are you currently responding to a custody case, or being investigated/supervised by the Guardian Council or other child protection program? |  |
| ASI | F6A | F_RelationshipProblemsPartner | In the last 30 days, did you have a relationship problem with partner? | ASI_FamilySocialProblem |
| ASI | F6B | F_RelationshipProblemsFamily | In the last 30 days, did you have a relationship problem with family? | ASI_FamilySocialProblem |
| ASI | F6C | F_RelationshipProblemsFriends | In the last 30 days, did you have a relationship problem with friends? | ASI_FamilySocialProblem |
| ASI | F7A | F_ArgumentPartner | In the last 30 days, did you have an arguments with partner? | ASI_FamilySocialProblem |
| ASI | F7B | F_ArgumentFamily | In the last 30 days, did you have an arguments with family? | ASI_FamilySocialProblem |
| ASI | F7C | F_ArgumentFriends | In the last 30 days, did you have an arguments with friend? | ASI_FamilySocialProblem |
| ASI | F8A | F_AlcDrugProblemaPartner | Does your partner have a current problem with alcohol or drug use? |  |
| ASI | F8B | F_AlcDrugProblemaFamily | Does your family have a current problem with alcohol or drug use? |  |
| ASI | F8C | F_AlcDrugProblemaFriends | Does your friends have a current problem with alcohol or drug use? |  |
| ASI | F9A | F_RelyPartner | If you need help, you can count on your partner? | ASI_FamilySocialSupport |
| ASI | F9B | F_RelyFamily | If you need help, you can count on your family? | ASI_FamilySocialSupport |
| ASI | F9C | F_RelyFriends | If you need help, you can count on your friends? | ASI_FamilySocialSupport |
| ASI | H1B | E_ControlledEnvironment_30Days | In the last 30 days, how many nights did you spend in a hospital, psychiatric inpatient or alcohol and/or drug treatment (inpatient) unit, prison or police station, sheltered boarding house or shelter for psychiatric patients, or therapeutic community? |  |
| ASI | H10 | E_HomelessShelter_Life | Have you ever stayed in a homeless shelter or on the street (in places like abandoned buildings, cars, parks, or squares) because you had nowhere else to stay? |  |
| ASI | H13B* | E_ControlledEnviromentTotal_30Days | Number of days in total living in a restricted/supervised housingor shelter situation? |  |
| ASI | H2B | E_UnitAlcoholDrug30Days | In the past 6 months, how many nights did you spend in aalcohol and/or drug treatment (inpatient) unit? |  |
| ASI | H3B | E_Hospital_30Days | In the past 6 months, how many nights did you spend in a hospital? | ASI_Medical |
| ASI | H4B | E_PsychiatricHospital_30Days | In the past 6 months, how many nights did you spend in a psychiatric inpatient unit? | ASI_Psychiatric |
| ASI | H5B | E_PoliceStationPrison_30Days | In the past 6 months, how many nights did you spend in a prison or police station? |  |
| ASI | H6B | E_TherapeuticCommunity_30Days | In the past 6 months, how many nights did you spend in a therapeutic community? |  |
| ASI | H7B | E_OtherControledEnvironment_30Days | In the past 6 months, how many nights did you spend in other controlled environment not mentioned yet? |  |
| ASI | H8B | E_HomelessShelter_30Days | In the past 6 months, how many nights did you spend in a shelter for homeless people? |  |
| ASI | H9B | E_Nostablearrangemen_30Days | How many nights did you spend on the street, or in places with abandoned buildings, cars, parks, or squares, because you had no other place to stay? |  |
| ASI | L1 | L_Arrested_Life | In your entire life, have you ever been in a prison or detained at a police station, even for a few hours? |  |
| ASI | L16 | L_TimesConvictedAdulthood | How many times have you been convicted of a crime committed after the age of 18? |  |
| ASI | L17B | L_TotalPeriodInJailAdulthood_Month | Since age 18, how much total time have you spent in jail or prison in months? |  |
| ASI | L18 | L_AdmissionPrompted | Was your admission for treatment court-ordered? |  |
| ASI | L19 | L_UnderInvestigation | Are you currently under investigation in a police investigation? |  |
| ASI | L2 | L_InJail_Life | In your entire life, have you ever been arrested? |  |
| ASI | L20 | L_SuspendedProcess | Are you currently on conditional suspension from any proceedings? |  |
| ASI | L21 | L_WatingSentence | Are you currently waiting for sentence? |  |
| ASI | L22 | L_OnParole | Are you currently on parole? |  |
| ASI | L23 | L_TherapeuticProgramJustice | Are you currently participating in a therapeutic justice program? |  |
| ASI | L25 | L_LegalTroubleBother_scl | How serious do you consider your current problems with criminal justice? | ASI_Legal |
| ASI | L26B | L_DrugTraffcking_30Days | In the last 30 days, did you sell or manufacture drugs? | ASI_Legal |
| ASI | L27B | L_Robbery_30Days | In the past 30 days, did you steal from anyone? | ASI_Legal |
| ASI | L28B | L_CrimeProperties_30Days | In the last 30 days, did you steal, steal, break in, defrauded, forged prescriptions or checks, destroyed property, or set fire to something? | ASI_Legal |
| ASI | L29B | L_ThreatenedAssaulted_30Days | In the past 30 days, have you threatened or assaulted anyone? | ASI_Legal |
| ASI | L30B | L_AnyOtherIllegalActivity_30Days | In the past 30 days, have you done anything else illegal? | ASI_Legal |
| ASI | L31B | L_CrimeIllegalActivity_30Days | In total, in the past 30 days, how many days did you do any of the above activities/things? | ASI_Legal |
| ASI | L32B | L_DrunkDriving_30Days | How many days in total did you drive under the influence of drugs or alcohol? |  |
| ASI | L5 | L_TimesConvictedUnder18 | How many times were you convicted of an offense before the age of 18? |  |
| ASI | L6 | L_TotalPeriodInJailUnder18_Month | Before the age of 18, how long did you spend total time locked up in detention centers or institutions for juvenile offenders? |  |
| ASI | M10 | M_Tuberculosis | Do you have tuberculosis? |  |
| ASI | M11 | M_Hepatitis | Do you have hepatitis? |  |
| ASI | M12 | M_CirrhosisLiverDisease | Do you have cirrhosis or any liver disease? |  |
| ASI | M13 | M_KidneyDisease | Do you have kidney disease? |  |
| ASI | M14 | M_LungDisease | Do you have lung disease? |  |
| ASI | M16 | M_Handicapped | Are you disable in any way? |  |
| ASI | M17* | M_PrescribedMedication_Yes | Dummy coded for having been prescribed medication for any medical conditions: Yes |  |
| ASI | M18 | M_MedPension | Have you ever requested or received any kind benefits for physical illness or disability? |  |
| ASI | M2 | M_CurrentPregnant | Are you currently pregnant? |  |
| ASI | M20 | M_MedProblems_30Days | How many days did you have physical or clinical symptoms or problems? | ASI_Medical |
| ASI | M21 | M_IncapacitatedMedProblems_30Days | How many days were you unable to carry out normal activities because of symptoms or medical/physical problems? | ASI_Medical |
| ASI | M22 | M_DiscomfortPhysicalPain_scl | How much discomfort or physical pain? | ASI_Medical |
| ASI | M23 | M_MedTroubBother_scl | How worried or troubled have you been about your physical health or any medical problems? | ASI_Medical |
| ASI | M24 | M_MedImportance_scl | At this time, how important is treatment (current or additional) to you for any medical or physical problem? | ASI_Medical |
| ASI | M25 | M_MedProbHospitalizations_Life | How many times in your life have you been hospitalized (at least one night) for physical or medical problems? |  |
| ASI | M26B | M_EmergencyServices_30Days | In the last 30 days, how many days did you use emergency services to treat a clinical problem? |  |
| ASI | M27B | M_MedProblemMedication_30Days | In the last 30 days, How many days have you taken prescribed medications for a physical illness?? |  |
| ASI | M28B | M_OutpatientService_30Days | In the last 30 days, How many days did you make outpatient or office visits with a doctor or health professional? |  |
| ASI | M3 | M_Hypertension | Do you have hypertension? |  |
| ASI | M4 | M_Diabetes | Do you have diabetes? |  |
| ASI | M5 | M_HeartDisease | Do you have any heart disease? |  |
| ASI | M6 | M_StrokeIschemia | Do you ever have a stroke of ischemia? |  |
| ASI | M7 | M_EpilepsySeizures | Do you have epilepsy or have you ever had any seizures? |  |
| ASI | M8 | M_Cancer | Do you have/ever had cancer? |  |
| ASI | M9 | M_HIV | Do you have HIV? |  |
| ASI | P1 | P_PsychHospitalizations | In your life, how many times have you been hospitalized for psychological/psychiatric problems? |  |
| ASI | P10A | P_AnxietyLife | Have you ever feel anxious, nervous, or worried most of the day (almost every day for at least 2 weeks at a time)? |  |
| ASI | P10B | P_Anxiety_30Days | In the last 30 days, | ASI_Psychiatric |
| ASI | P10C | P_AnxietyLastTime | How many days ago were you anxious, nervous or worried most of the day (nearly every day for at least 2 weeks in a row)? |  |
| ASI | P11A | P_Hallucinations_Life | Have you ever experience hallucinations? |  |
| ASI | P11B | P_Hallucinations_30Days | In the last 30 days, did you experience hallucinations? | ASI_Psychiatric |
| ASI | P11C | P_HallucinationsLastTime | How many days ago did you experience hallucinations? |  |
| ASI | P12A | P_ThinkingAttention_Life | Have you ever had trouble thinking/concentrating, understanding or remembering, to the point where it caused you problems? |  |
| ASI | P12B | P_ThinkingAttention_30Days | In the last 30 days, did you have trouble thinking/concentrating, understanding or remembering, to the point where it caused you problems? | ASI_Psychiatric |
| ASI | P12C | P_ThinkingAttentionLastTime | How many days ago did you have trouble thinking/concentrating, understanding or remembering, to the point where it caused you problems? |  |
| ASI | P13A | P_Impulsivity_Life | (Since age 18) Have you had difficulty controlling your temper, or your impulses to hit or hurt someone? |  |
| ASI | P13B | P_Impulsivity_30Days | In the last 30 days, did you experience difficulty in controlling your temper, or your impulses to hit or hurt someone? | ASI_Psychiatric |
| ASI | P13C | P_ImpulsivityLastTime | How many days ago have you had difficulty controlling your temper, or your impulses to hit or hurt someone? |  |
| ASI | P14A | P_Aggressivity_Life | (Since age 18) Have you pushed, hit, threw, or used a weapon against someone? |  |
| ASI | P14B | P_Aggressivity_30Days | In the last 30 days, did you pushed, hit, threw, or used a weapon against someone? | ASI_Psychiatric |
| ASI | P14C | P_AggressivityLastTime | How many days ago have you pushed, hit, threw, or used a weapon against someone? |  |
| ASI | P15A | P_SuicideThough_Life | Have you ever had serious thoughts about suicide (or killing yourself)? |  |
| ASI | P15B | P_SuicideThough_30Days | In the last 30 days, have you had serious thoughts about suicide (or killing yourself)? | ASI_Psychiatric |
| ASI | P15C | P_SuicideThough | How many days ago have you had serious thoughts about suicide (or killing yourself)? |  |
| ASI | P16A | P_SuicideAttempt_Life | Have you ever attempted suicide (kill yourself)? |  |
| ASI | P16B | P_SuicideAttempt_30Days | In the last 30 days, have you had attempted suicide (kill yourself)? | ASI_Psychiatric |
| ASI | P16C | P_SuicideAttemptLastTime | How many days ago have you had attempted suicide (kill yourself)? |  |
| ASI | P17A | P_OtherPsychProblemLastTime_Life | Have you ever experienced another psychological or psychiatric problem not mentioned yet? |  |
| ASI | P17B | P_OtherPsychProblem_30Days | In the last 30 days, have you experienced another psychological or psychiatric problem not mentioned yet? |  |
| ASI | P17C | P_OtherPsychProblemLastTime | How many days ago have you experienced another psychological or psychiatric problem not mentioned yet? |  |
| ASI | P18 | P_PsychProblems_30Days | In the last 30 days, how many days have you had these psychological or psychiatric problems? | ASI_Psychiatric |
| ASI | P19 | P_PsychDisable_30Days | In the last 30 days, how many days were you unable to carry out your normal activities because of psychological problems or psychiatric symptoms? | ASI_Psychiatric |
| ASI | P2 | P_PsychMedEverPrescribed | In your life, have you ever been prescribed medication to treat psychological/psychiatric problems? |  |
| ASI | P20 | P_PsychTroubleBother_scl | How worried or troubled have you been by these psychological or psychiatric problems? | ASI_Psychiatric |
| ASI | P21 | P_PsychImportance_scl | At this time, how important is treatment (current or additional) for psychological/psychiatric problems to you? | ASI_Psychiatric |
| ASI | P3B | P_PsychMed_30Days | In the last 30 days, how many days have you taken medications to treat psychological/psychiatric problems? |  |
| ASI | P4 | P_NumberOutpatientTreat | In your lifetime, how many outpatient appointments have you had for psychological/psychiatric problems? |  |
| ASI | P5B | P_Outpatients_30Days | In the last 30 days, how many outpatient or office visits have you had? |  |
| ASI | P7 | P_PsychPension | Do you currently receive a pension (or benefit) for psychological impairment/disability? |  |
| ASI | P8B | P_SleepProblems_30Days | In the last 30 days, did you have trouble falling asleep, staying asleep*, or waking up too early? | ASI_Psychiatric |
| ASI | P9A | P_Depressed_Life | Have you ever felt depressed or down most of the day (nearly every day for at least 2 weeks at a time)? |  |
| ASI | P9B | P_Depressed_30Days | In the last 30 days, have you felt depressed or down most of the day (nearly every day for at least 2 weeks at a time)? | ASI_Psychiatric |
| ASI | P9C | P_DepressedLastTime | How many days ago have you felt depressed or down most of the day (nearly every day for at least 2 weeks at a time)? |  |
| ASI | TS1 | ASI_Drugs | ASI Score for drug related problems |  |
| ASI | TS2 | ASI_FamilyChild | ASI Score for family and child related problems |  |
| ASI | TS3 | ASI_Alcohol | ASI Score for alcohol related problems |  |
| ASI | TS4 | ASI_Psychiatric | ASI Score for psychiatric related problems |  |
| ASI | TS5 | ASI_Medical | ASI Score for medical related problems |  |
| ASI | TS6 | ASI_Legal | ASI Score for legal related problems |  |
| ASI | TS7 | ASI_Employement | ASI Score for employment related problems |  |
| ASI | TS8 | ASI_FamilySocialSupport | ASI Score for family and social support related problems |  |
| ASI | TS9 | ASI_FamilySocialProblem | ASI Score for family and social related problems |  |
| CTQ | na. | T_SexualAbuse_CTQ | CTQ Sexual abuse subscore |  |
| CTQ | na. | T_EmotionalAbuse_CTQ | CTQ Emotional abuse subscore |  |
| CTQ | na. | T_EmotionalNeglect_CTQ | CTQ Emotional neglect subscore |  |
| CTQ | na. | T_PhysicalAbuse_CTQ | CTQ Physical abuse subscore |  |
| CTQ | na. | T_PhysicalNeglect_CTQ | CTQ Physical neglect subscore |  |
| CTQ | na. | CTQ Total score | CTQ Total score |  |
| CTQ | na. | PA_DIC_LG | CTQ Physical abuse dichotomic |  |
| CTQ | na. | PN_DIC_LG | CTQ Physical neglect dichotomic |  |
| CTQ | na. | SA_DIC_LG | CTQ Sexual abuse dichotomic |  |
| CTQ | na. | EA_DIC_LG | CTQ Emotional abuse dichotomic |  |
| CSSA | na. | CSSA Score first week of treatment | CSSA Score first week of treatment |  |
| CSSA | na. | CSSA Score third week of treatment | CSSA Score third week of treatment (Not a feature, since it was used to generate the outcome) |  |

Note: Original variable name represents the codes used in ASI-6. ASI Score indicates if a given variable was used to generate the ASI composite scores. ASI - Addiction Severity Index, 6 edition; CSSA - Cocaine Selective Severity Assessment; CTQ - Childhood Trauma Questionnaire.
