# Supplementary material for: Examining predictors of cocaine withdrawal syndrome at the end of detoxification treatment in women with cocaine use disorder
Source: J Psychiatr Res. 2024 Jan;169:247–56. doi: 10.1016/j.jpsychires.2023.11.043 (PMC10805009; doi:10.1016/j.jpsychires.2023.11.043)
Supplement: Supplementary file 4 — Multimedia component 4 [file mmc4.docx]

|  | **Non-severe withdrawal** | **Severe withdrawal** | **p<0.05** |
| --- | --- | --- | --- |
| **Variables** | Mean (SD) | Mean (SD) | not sig |
| ASI.6.Drug.related.problems.score | 50.14 (4.22) | 51.80 (5.24) | not sig |
| ASI_FamilyChild | 56.76 (10.19) | 56.61 (9.87) | not sig |
| ASI_Alcohol | 49.58 (5.91) | 49.73 (6.10) | not sig |
| ASI.6.Psychiatric.related.problems.score | 48.21 (6.59) | 51.59 (7.16) | sig |
| ASI_Medical | 45.32 (5.54) | 45.65 (5.02) | not sig |
| ASI_Legal | 48.27 (4.57) | 50.27 (5.99) | sig |
| ASI_Employement | 39.39 (2.62) | 40.29 (3.21) | not sig |
| ASI_FamilySocialSupport | 67.65 (13.05) | 68.15 (11.72) | not sig |
| ASI.6.Family.and.social.related.problems.score | 61.40 (14.67) | 61.43 (15.23) | not sig |
| ASI.6.Anytime.in.controlled.environment.last.30.days.before.treatment | 6.14 (5.74) | 5.94 (6.62) | not sig |
| E_UnitAlcoholDrug30Days | 5.97 (5.02) | 5.63 (6.26) | not sig |
| E_Hospital_30Days | 0.24 (1.32) | 0.20 (0.93) | not sig |
| E_PsychiatricHospital_30Days | 0.20 (1.81) | 0.01 (0.08) | not sig |
| E_PoliceStationPrison_30Days | 0.06 (0.59) | 0.08 (0.84) | not sig |
| E_TherapeuticCommunity_30Days | 0.67 (3.60) | 0.01 (0.08) | not sig |
| E_OtherControledEnvironment_30Days | 0.03 (0.34) | 0.03 (0.34) | not sig |
| E_HomelessShelter_30Days | 0.08 (0.85) | 0.00 (0.00) | not sig |
| E_Nostablearrangemen_30Days | 1.63 (4.93) | 1.85 (5.55) | not sig |
| E_HomelessShelter_Life | 0.02 (0.14) | 0.18 (0.39) | sig |
| E_ControlledEnviromentTotal_30Days | 7.22 (7.09) | 5.95 (6.39) | not sig |
| M_CurrentPregnant | 0.04 (0.20) | 0.27 (0.44) | sig |
| M_Hypertension | 0.05 (0.22) | 0.26 (0.44) | sig |
| M_Diabetes | 0.04 (0.18) | 0.29 (0.45) | sig |
| M_HeartDisease | 0.04 (0.20) | 0.29 (0.45) | sig |
| M_StrokeIschemia | 0.05 (0.22) | 0.29 (0.45) | sig |
| M_EpilepsySeizures | 0.05 (0.22) | 0.27 (0.45) | sig |
| M_Cancer | 0.05 (0.22) | 0.30 (0.46) | sig |
| M_HIV | 0.05 (0.22) | 0.30 (0.46) | sig |
| M_Tuberculosis | 0.05 (0.22) | 0.29 (0.45) | sig |
| M_Hepatitis | 0.05 (0.22) | 0.27 (0.45) | sig |
| M_CirrhosisLiverDisease | 0.05 (0.22) | 0.30 (0.46) | sig |
| M_KidneyDisease | 0.05 (0.22) | 0.27 (0.45) | sig |

| M_LungDisease | 0.04 (0.18) | 0.18 (0.38) | sig |
| --- | --- | --- | --- |
| M_Handicapped | 0.05 (0.22) | 0.19 (0.39) | sig |
| M_PrescribedMedication_Yes | 0.04 (0.20) | 0.17 (0.38) | sig |
| M_MedPension | 0.04 (0.20) | 0.27 (0.44) | sig |
| M_MedProblems_30Days | 6.53 (9.94) | 5.45 (9.14) | not sig |
| M_IncapacitatedMedProblems_30Days | 2.59 (5.87) | 2.42 (6.28) | not sig |
| M_MedProbHospitalizations_Life | 3.23 (8.71) | 3.15 (8.81) | not sig |
| M_EmergencyServices_30Days | 0.54 (2.96) | 0.23 (1.02) | not sig |
| M_MedProblemMedication_30Days | 1.11 (3.35) | 1.07 (3.82) | not sig |
| M_OutpatientService_30Days | 0.38 (1.43) | 0.11 (0.41) | not sig |
| E_CurrentStudying_FullTime | 0.03 (0.17) | 0.02 (0.14) | not sig |
| E_CurrentStudying_No | 0.97 (0.17) | 0.97 (0.17) | not sig |
| E_DriverLicense | 0.92 (0.27) | 0.93 (0.26) | not sig |
| E_OwnVehicle | 0.18 (0.38) | 0.13 (0.34) | not sig |
| E_LaborStudyingDifficultVehicle | 0.23 (0.42) | 0.20 (0.40) | not sig |
| E_WriteRead | 0.87 (0.34) | 0.84 (0.37) | not sig |
| E_PaidWork_30Days | 2.55 (5.85) | 4.20 (8.06) | not sig |
| E_GrossIncome_PerMonth_Adjusted | 375.49 (618.19) | 763.22 (1848.17) | not sig |
| E_EstimatedTotalGrossIncome_Yrs_PPPadj | 158.97 (261.72) | 323.12 (782.46) | not sig |
| E_EmployProbs_30Days | 0.13 (0.75) | 0.54 (2.73) | not sig |
| E_FinancedHousingAssistance | 0.04 (0.20) | 0.11 (0.31) | not sig |
| E_MoneyPension_30Days_Adjusted | 133.30 (422.94) | 339.73 (1553.55) | not sig |
| E_MoneyWelfare_30Days_Adjusted | 86.65 (183.48) | 102.61 (233.11) | not sig |
| E_AdditionalMoneyWelfare_30Days_Adjusted | 29.39 (173.52) | 71.39 (375.22) | not sig |
| E_MoneyAlimony_30Days_Adjusted | 39.23 (160.08) | 54.93 (270.22) | not sig |
| E_MoneyOtherSource_30Days_Adjusted | 86.93 (260.78) | 194.56 (884.90) | not sig |
| E_EmploymentMainSource | 2.96 (0.73) | 2.49 (1.02) | sig |
| E_RetirementMainSource | 2.87 (0.70) | 2.45 (0.98) | sig |
| E_HealthPensionMainSource | 2.89 (0.72) | 2.43 (0.97) | sig |
| E_UnemploymentInsurMainSource | 2.83 (0.66) | 2.37 (0.96) | sig |
| E_PublicWelfareMainSource | 3.01 (0.81) | 2.54 (1.09) | sig |
| E_AlimonyMainSource | 2.86 (0.69) | 2.41 (0.95) | sig |
| E_FriendsFamilyMainSource | 3.27 (0.83) | 2.79 (1.08) | sig |
| L_IlegalMainSource | 2.86 (0.65) | 2.49 (0.97) | sig |

| E_InstitutionalizedMainSource | 2.83 (0.64) | 2.35 (0.97) | sig |
| --- | --- | --- | --- |
| E_Bankruptcy | 0.05 (0.22) | 0.06 (0.23) | not sig |
| E_PersonalDebt | 0.30 (0.46) | 0.20 (0.40) | not sig |
| E_CurrentPersonalDebt | 0.42 (0.50) | 0.42 (0.50) | not sig |
| E_EnoughtMoneyLiving | 0.04 (0.18) | 0.19 (0.39) | sig |
| ASI.6.Number.of.drug.alcohol.related.treatments.underwent | 3.25 (3.07) | 3.85 (4.14) | not sig |
| D_AlcDrugsDetox | 2.91 (3.15) | 3.27 (3.74) | not sig |
| D_AlcDrugsOutpatient_6Monhts | 7.46 (21.09) | 8.50 (29.57) | not sig |
| D_AlcDrugsOutpatient_30Days | 2.44 (5.79) | 1.85 (5.38) | not sig |
| D_AlcDrugsPrescribedMed_30Days | 8.20 (10.78) | 7.26 (10.76) | not sig |
| D_AA_30Days | 0.25 (1.11) | 1.04 (4.57) | not sig |
| D_AALongestPeriod_Months | 1.33 (5.66) | 4.30 (26.65) | not sig |
| D_AlcRegularUse_Yrs | 4.65 (7.85) | 3.73 (6.86) | not sig |
| D_AlcRegularAbuse_Yrs | 3.85 (7.15) | 3.43 (6.58) | not sig |
| D_Alc50DaysMore | 0.39 (0.49) | 0.40 (0.49) | not sig |
| D_AlcFrequencyASI_week | 2.88 (2.97) | 2.72 (3.04) | not sig |
| D_AlcUse_30Days | 7.27 (10.04) | 8.06 (10.52) | not sig |
| D_AlcAbuse_30Days | 6.20 (9.45) | 6.69 (9.98) | not sig |
| D_AlcMoneySpent | 55.49 (136.13) | 69.71 (172.41) | not sig |
| D_AlcUncontrolledSymtom | 0.25 (0.43) | 0.30 (0.46) | not sig |
| D_AlcMedPsychSymtom | 0.30 (0.46) | 0.30 (0.46) | not sig |
| D_AlcEverCrave | 0.19 (0.39) | 0.26 (0.44) | not sig |
| D_AlcProblems_30Days | 3.09 (7.00) | 3.98 (8.31) | not sig |
| D_Cannabis50DaysMore | 0.52 (0.50) | 0.75 (0.43) | sig |
| ASI.6.Ratio.between.age.and.years.of.regular.use.of.cannabis | 0.17 (0.22) | 0.24 (0.24) | not sig |
| D_Sedative50DaysMore | 0.12 (0.33) | 0.18 (0.39) | not sig |
| D_SedativesRegAbuseRatio | 0.02 (0.10) | 0.05 (0.13) | not sig |
| D_Cocaine50DaysMore | 0.70 (0.46) | 0.66 (0.47) | not sig |
| D_CocaineRegAbuseRatio | 0.16 (0.19) | 0.18 (0.19) | not sig |
| D_Crack50DaysMore | 0.63 (0.48) | 0.79 (0.41) | not sig |
| D_CrackRegAbuseRatio | 0.16 (0.18) | 0.18 (0.18) | not sig |
| D_Stimulants50DaysMore | 0.01 (0.08) | 0.02 (0.14) | not sig |
| D_StimulantsAsTreatment | 0.01 (0.08) | 0.01 (0.08) | not sig |
| D_Hallucin50DaysMore | 0.01 (0.12) | 0.01 (0.12) | not sig |

| D_Opioid50DaysMore | 0.01 (0.12) | 0.00 (0.00) | not sig |
| --- | --- | --- | --- |
| D_Inhalant50DaysMore | 0.13 (0.33) | 0.17 (0.38) | not sig |
| D_InhalantAsTreatment | 0.00 (0.00) | 0.04 (0.20) | not sig |
| D_NicotineUse_Life | 0.95 (0.22) | 0.91 (0.29) | not sig |
| D_Nicotine_Yrs | 2.92 (4.46) | 2.23 (4.09) | not sig |
| D_Nicotine50DaysMore | 0.86 (0.35) | 0.87 (0.34) | not sig |
| D_Nicotine_30Days | 25.32 (10.19) | 23.58 (11.63) | not sig |
| D_NicotineCraving | 5.18 (4.29) | 4.51 (4.62) | not sig |
| ASI.6.Ratio.between.age.and.years.of.regular.use.of.nicotine | 0.40 (0.27) | 0.44 (0.27) | not sig |
| D_NicotineDailyUse_Yrs | 15.04 (10.52) | 14.39 (10.07) | not sig |
| D_DrugFrequencyASI_week | 1.41 (2.59) | 1.33 (2.59) | not sig |
| ASI.6.Days.consuming.drugs.in.the.last.month.before.detoxification | 14.92 (9.54) | 18.11 (9.64) | not sig |
| D_DrugUncontrolledSymtom | 0.70 (0.46) | 0.81 (0.39) | not sig |
| D_DrugMedPsychSymtom | 0.75 (0.44) | 0.69 (0.46) | not sig |
| ASI.6.Drug.related.craving.symptoms.last.30.days.before.treatment | 0.42 (0.50) | 0.73 (0.44) | sig |
| D_DrugProblems_30Days | 12.73 (11.17) | 15.00 (11.99) | not sig |
| D_GamblingProblems | 0.06 (0.23) | 0.04 (0.18) | not sig |
| D_GamblingProblems_30Days | 0.23 (1.64) | 0.13 (1.03) | not sig |
| L_Arrested_Life | 0.34 (0.47) | 0.33 (0.47) | not sig |
| L_InJail_Life | 0.21 (0.41) | 0.21 (0.41) | not sig |
| L_TimesConvictedUnder18 | 0.04 (0.20) | 0.03 (0.17) | not sig |
| L_TotalPeriodInJailUnder18_Month | 0.15 (1.15) | 0.27 (2.13) | not sig |
| L_TimesConvictedAdulthood | 0.18 (0.87) | 0.22 (1.16) | not sig |
| L_TotalPeriodInJailAdulthood_Month | 0.42 (1.41) | 0.29 (1.25) | not sig |
| L_AdmissionPrompted | 0.08 (0.27) | 0.06 (0.24) | not sig |
| L_UnderInvestigation | 0.03 (0.17) | 0.04 (0.20) | not sig |
| L_SuspendedProcess | 0.01 (0.08) | 0.00 (0.00) | not sig |
| L_WatingSentence | 0.06 (0.23) | 0.06 (0.23) | not sig |
| L_OnParole | 0.02 (0.14) | 0.01 (0.12) | not sig |
| L_TherapeuticProgramJustice | 0.01 (0.12) | 0.01 (0.08) | not sig |
| L_DrugTraffcking_30Days | 0.18 (1.62) | 0.36 (2.25) | not sig |
| L_Robbery_30Days | 0.21 (1.32) | 0.39 (2.90) | not sig |
| L_CrimeProperties_30Days | 0.23 (1.82) | 0.80 (4.14) | not sig |
| L_AnyOtherIllegalActivity_30Days | 0.23 (2.16) | 1.10 (4.57) | not sig |

| L_CrimeIllegalActivity_30Days | 0.32 (2.28) | 1.12 (4.27) | not sig |
| --- | --- | --- | --- |
| L_DrunkDriving_30Days | 0.02 (0.14) | 0.42 (3.15) | not sig |
| F_RomanticSexualRelationship | 0.65 (0.48) | 0.73 (0.45) | not sig |
| F_CloseFriends | 1.68 (3.30) | 1.83 (5.75) | not sig |
| F_TimeTogheterPartner | 0.61 (0.49) | 0.71 (0.45) | not sig |
| F_TimeTogheterFamily | 0.72 (0.45) | 0.70 (0.46) | not sig |
| F_TimeTogheterFriends | 0.36 (0.48) | 0.34 (0.47) | not sig |
| F_AnyContactPartner | 0.46 (0.50) | 0.39 (0.49) | not sig |
| F_AnyContactFamily | 0.55 (0.50) | 0.58 (0.50) | not sig |
| F_AnyContactFriends | 0.29 (0.45) | 0.25 (0.44) | not sig |
| F_ShareFeelingsPartner | 0.43 (0.50) | 0.46 (0.50) | not sig |
| F_ShareFeelingsFamily | 0.48 (0.50) | 0.49 (0.50) | not sig |
| F_ShareFeelingsFriends | 0.27 (0.44) | 0.25 (0.43) | not sig |
| F_RelationshipProblemsPartner | 0.37 (0.49) | 0.42 (0.49) | not sig |
| F_RelationshipProblemsFamily | 0.35 (0.48) | 0.34 (0.47) | not sig |
| F_RelationshipProblemsFriends | 0.05 (0.22) | 0.10 (0.30) | not sig |
| F_ArgumentPartner | 0.42 (0.49) | 0.45 (0.50) | not sig |
| F_ArgumentFamily | 0.41 (0.49) | 0.35 (0.48) | not sig |
| F_ArgumentFriends | 0.07 (0.26) | 0.08 (0.28) | not sig |
| F_AlcDrugProblemaPartner | 0.32 (0.47) | 0.37 (0.48) | not sig |
| F_AlcDrugProblemaFamily | 0.36 (0.48) | 0.37 (0.49) | not sig |
| F_AlcDrugProblemaFriends | 0.17 (0.38) | 0.17 (0.38) | not sig |
| F_RelyPartner | 0.54 (0.50) | 0.58 (0.50) | not sig |
| F_RelyFamily | 0.80 (0.40) | 0.73 (0.45) | not sig |
| F_RelyFriends | 0.40 (0.49) | 0.32 (0.47) | not sig |
| F_JudicianlRemoval | 0.05 (0.22) | 0.04 (0.18) | not sig |
| F_PhysicalAgressionCloseFriends | 0.23 (0.42) | 0.32 (0.47) | not sig |
| F_SomeoneElseToRely | 0.35 (0.48) | 0.35 (0.48) | not sig |
| F_DifficExpressingEmotions | 0.63 (0.48) | 0.61 (0.49) | not sig |
| F_SocialDisconfort | 0.49 (0.50) | 0.47 (0.50) | not sig |
| F_RelationshipImportance | 0.76 (0.43) | 0.81 (0.39) | not sig |
| F_AttendedReliousGroup | 0.28 (0.45) | 0.26 (0.44) | not sig |
| F_VoluntaryWork | 0.06 (0.23) | 0.07 (0.26) | not sig |
| F_DifficSpendingFreeTime | 0.54 (0.50) | 0.58 (0.49) | not sig |

| T_PhysicallyAssaultedSomeoneKnown | 0.56 (0.50) | 0.69 (0.46) | not sig |
| --- | --- | --- | --- |
| T_Raped | 0.35 (0.48) | 0.48 (0.50) | not sig |
| T_ViolentCrimeBeaten | 0.25 (0.43) | 0.25 (0.43) | not sig |
| T_DeathRisk | 0.34 (0.47) | 0.31 (0.46) | not sig |
| T_WitnessExtremeViolence | 0.54 (0.50) | 0.49 (0.50) | not sig |
| F_OpenCustodyProceedings | 0.17 (0.38) | 0.20 (0.40) | not sig |
| F_ChildrenLivingTogether_30Days | 0.91 (1.32) | 0.96 (2.28) | not sig |
| F_OtherChildLivingTogether_30Days | 0.16 (0.37) | 0.14 (0.35) | not sig |
| F_ChildcareAssistance | 0.35 (0.48) | 0.34 (0.47) | not sig |
| F_EverCustodyProcess | 0.30 (0.46) | 0.32 (0.47) | not sig |
| F_ChildrenEverProtected | 0.14 (0.35) | 0.13 (0.34) | not sig |
| F_SuspendedRightsChildren | 0.14 (0.35) | 0.19 (0.39) | not sig |
| F_UnderCustodyProcess | 0.11 (0.31) | 0.15 (0.36) | not sig |
| P_PsychHospitalizations | 1.08 (4.19) | 0.70 (2.68) | not sig |
| P_PsychMedEverPrescribed | 0.46 (0.50) | 0.44 (0.50) | not sig |
| P_PsychMed_30Days | 1.46 (5.13) | 1.46 (4.76) | not sig |
| P_NumberOutpatientTreat | 1.20 (1.53) | 1.14 (1.47) | not sig |
| P_Outpatients_30Days | 0.30 (0.78) | 0.52 (2.22) | not sig |
| P_PsychPension | 0.05 (0.22) | 0.08 (0.28) | not sig |
| P_SleepProblems_30Days | 0.86 (0.85) | 0.97 (0.80) | not sig |
| P_Depressed_Life | 0.92 (0.74) | 0.91 (0.64) | not sig |
| P_Depressed_30Days | 0.80 (0.87) | 0.82 (0.78) | not sig |
| P_DepressedLastTime | 2.04 (5.07) | 0.87 (3.11) | not sig |
| P_AnxietyLife | 0.78 (0.76) | 0.86 (0.66) | not sig |
| P_Anxiety_30Days | 0.70 (0.86) | 0.79 (0.78) | not sig |
| P_AnxietyLastTime | 1.13 (3.61) | 0.66 (3.06) | not sig |
| P_Hallucinations_Life | 0.67 (0.87) | 0.80 (0.83) | not sig |
| P_Hallucinations_30Days | 0.47 (0.81) | 0.50 (0.78) | not sig |
| P_HallucinationsLastTime | 2.72 (5.77) | 2.37 (5.32) | not sig |
| P_ThinkingAttention_Life | 0.71 (0.82) | 0.68 (0.71) | not sig |
| P_ThinkingAttention_30Days | 0.60 (0.84) | 0.63 (0.77) | not sig |
| P_ThinkingAttentionLastTime | 1.61 (3.92) | 1.37 (3.31) | not sig |
| P_Impulsivity_Life | 0.58 (0.73) | 0.58 (0.69) | not sig |
| P_Impulsivity_30Days | 0.42 (0.75) | 0.42 (0.69) | not sig |

| P_ImpulsivityLastTime | 1.62 (4.25) | 2.37 (5.62) | not sig |
| --- | --- | --- | --- |
| P_Aggressivity_Life | 0.58 (0.77) | 0.62 (0.71) | not sig |
| P_Aggressivity_30Days | 0.26 (0.64) | 0.36 (0.69) | not sig |
| P_AggressivityLastTime | 0.90 (3.43) | 2.46 (6.11) | not sig |
| P_SuicideThough_Life | 0.04 (0.20) | 0.41 (0.75) | sig |
| P_SuicideThough_30Days | 0.53 (0.65) | 0.87 (0.76) | sig |
| P_SuicideThough | 2.35 (5.36) | 2.98 (6.20) | not sig |
| P_SuicideAttempt_Life | 0.04 (0.20) | 0.42 (0.73) | sig |
| P_SuicideAttempt_30Days | 0.04 (0.18) | 0.29 (0.53) | sig |
| P_SuicideAttemptLastTime | 1.43 (4.27) | 1.44 (4.70) | not sig |
| P_OtherPsychProblemLastTime_Life | 0.23 (0.56) | 0.15 (0.40) | not sig |
| P_OtherPsychProblem_30Days | 0.15 (0.52) | 0.08 (0.35) | not sig |
| P_OtherPsychProblemLastTime | 0.86 (4.09) | 2.58 (8.41) | not sig |
| P_PsychProblems_30Days | 5.04 (7.67) | 5.04 (7.50) | not sig |
| CTQ.Sexual.abuse.subscore | 7.02 (3.98) | 8.77 (5.52) | sig |
| CTQ.Emotional.abuse.subscore | 10.43 (5.34) | 12.58 (5.76) | sig |
| CTQ.Emotional.neglect.subscore | 10.25 (5.55) | 11.80 (5.94) | not sig |
| CTQ.Physical.abuse.subscore | 9.30 (5.37) | 10.15 (5.35) | not sig |
| CTQ.Physical.neglect.subscore | 8.42 (3.99) | 8.92 (4.30) | not sig |
| CTQ.Total.score | 45.42 (17.80) | 52.22 (21.16) | not sig |
| CTQ.PA_DIC_LG | 1.66 (1.04) | 2.11 (1.19) | sig |
| CTQ.PN_DIC_LG | 2.05 (1.17) | 2.48 (1.22) | sig |
| CTQ.SA_DIC_LG | 0.24 (0.43) | 0.42 (0.49) | sig |
| CTQ.EA_DIC_LG | 0.31 (0.46) | 0.49 (0.50) | sig |
| D_HeroinUse_Life | 0.01 (0.12) | 0.01 (0.12) | not sig |
| D_OpioidUse_Life | 0.03 (0.17) | 0.00 (0.00) | not sig |
| D_StimulantsUse_Life | 0.05 (0.22) | 0.06 (0.24) | not sig |
| D_HallucinUse_Life | 0.09 (0.29) | 0.11 (0.31) | not sig |
| D_SedativeUse_Life | 0.21 (0.41) | 0.31 (0.46) | not sig |
| D_InhalantUse_Life | 0.37 (0.49) | 0.40 (0.49) | not sig |
| D_CannabisUse_Life | 0.87 (0.33) | 0.89 (0.31) | not sig |
| D_CrackCocaineUse_Life | 0.98 (0.14) | 0.97 (0.17) | not sig |
| D_CrackUse_Life | 0.77 (0.42) | 0.86 (0.35) | not sig |
| D_CocaineUse_Life | 0.90 (0.30) | 0.87 (0.34) | not sig |

| D_Heroin_30Days | 0.01 (0.08) | 0.21 (2.52) | not sig |
| --- | --- | --- | --- |
| D_Opioid_30Days | 0.07 (0.84) | 0.00 (0.00) | not sig |
| D_Stimulants_30Days | 0.00 (0.00) | 0.35 (2.78) | not sig |
| D_Hallucin_30Days | 0.01 (0.12) | 0.00 (0.00) | not sig |
| D_Sedative_30Days | 2.92 (8.30) | 3.87 (9.23) | not sig |
| D_Inhalant_30Days | 0.06 (0.46) | 0.42 (2.95) | not sig |
| D_Cannabis_30Days | 8.39 (12.86) | 8.15 (12.63) | not sig |
| ASI.6.Days.consuming.crack.or.cocaine.the.last.month.before.detoxification | 19.42 (11.91) | 21.87 (11.21) | not sig |
| ASI.6.Days.consuming.crack.the.last.month.before.detoxification | 14.32 (13.48) | 18.88 (12.94) | not sig |
| D_Cocaine_30Days | 7.45 (11.32) | 7.24 (11.63) | not sig |
| D_Opioid_Yrs | 0.01 (0.17) | 0.00 (0.00) | not sig |
| D_Stimulants_Yrs | 0.00 (0.00) | 0.16 (1.29) | not sig |
| D_Hallucin_Yrs | 0.09 (1.01) | 0.04 (0.30) | not sig |
| D_Sedative_Yrs | 0.58 (2.58) | 0.82 (2.65) | not sig |
| D_Inhalant_Yrs | 0.33 (1.26) | 0.37 (1.26) | not sig |
| D_Cannabis_Yrs | 2.44 (4.01) | 3.19 (4.60) | not sig |
| D_CrackCocaine_Yrs | 6.17 (5.28) | 6.92 (5.08) | not sig |
| D_Crack_Yrs | 4.44 (5.29) | 4.93 (5.07) | not sig |
| D_Cocaine_Yrs | 3.37 (4.30) | 3.96 (4.76) | not sig |
| CSSA.Score.first.week.of.treatment | 32.16 (18.14) | 48.33 (17.99) | sig |
| CSSA.Score.before.treatment.discharge (Not a feature) | 12.49 (5.61) | 42.05 (16.29) | sig |

Note: Group comparisons performed by t-test, mann-whitney, or chi-squared test when appropriate. SD – standard deviation. ASI - Addiction Severity Index, 6 edition; CSSA - Cocaine Selective Severity Assessment; CTQ - Childhood Trauma Questionnaire.
