# Supplementary material for: Examining predictors of cocaine withdrawal syndrome at the end of detoxification treatment in women with cocaine use disorder
Source: J Psychiatr Res. 2024 Jan;169:247–56. doi: 10.1016/j.jpsychires.2023.11.043 (PMC10805009; doi:10.1016/j.jpsychires.2023.11.043)
Supplement: Multimedia component 5 [file mmc5.docx]

| Algorithm | Hyperparameter | Search Space |
| --- | --- | --- |
| KNN | n_neighbors | 1, 6, 11, 16, 21, 26 |
| KNN | weights | "uniform", "distance" |
| KNN | leaf_size | 1, 7, 13, 19, 25, 31 |
| KNN | p | 1, 2 |
| SVM | gamma | "scale", "auto" |
| SVM | C | 0.01, 0.06, 0.11, 0.16, 0.21, 0.26, 0.31, 0.36, 0.41, 0.46, 0.51, 0.56, 0.61, 0.66, 0.71, 0.76, 0.81, 0.86, 0.91, 0.96 |
| SVM | kernel | "linear", "rbf" |
| Log Reg | penalty | "elasticnet" |
| Log Reg | C | 0.01, 0.06, 0.11, 0.16, 0.21, 0.26, 0.31, 0.36, 0.41, 0.46, 0.51, 0.56, 0.61, 0.66, 0.71, 0.76, 0.81, 0.86, 0.91, 0.96 |
| Log Reg | l1_ratio | 0.1, 0.3, 0.5, 0.7, 0.9 |
| Log Reg | solver | "saga" |
| Log Reg | fit_intercept | True, False |
| Light GBM | n_estimators | 50, 100 |
| Light GBM | learning_rate | 0.005, 0.01, 0.1, 0.3 |
| Light GBM | num_leaves | 5, 10, 20 |
| Light GBM | min_child_samples | 5, 10, 20 |
| Light GBM | max_bin | 20, 50 |
| NB | var_smoothing | 150 equally spaced steps between 0 and 10^-11 |
| RF | min_samples_leaf | 1, 3, 5, 7, 9, 11 |
| RF | criterion | "gini", "entropy" |
| RF | n_estimators | 10, 30, 50, 70, 90 |
| RF | bootstrap | True, False |
