# Supplementary material for: Examining predictors of cocaine withdrawal syndrome at the end of detoxification treatment in women with cocaine use disorder
Source: J Psychiatr Res. 2024 Jan;169:247–56. doi: 10.1016/j.jpsychires.2023.11.043 (PMC10805009; doi:10.1016/j.jpsychires.2023.11.043)
Supplement: Multimedia component 6 [file mmc6.docx]

| Classification Algorithm | Outer CV Round | Hyperparameter | Value |
| --- | --- | --- | --- |
| Logistic Regression | CV Round 1 | penalty | "elasticnet" |
| Logistic Regression | CV Round 1 | C | 0,01 |
| Logistic Regression | CV Round 1 | l1_ratio | 0,9 |
| Logistic Regression | CV Round 1 | solver | "saga" |
| Logistic Regression | CV Round 1 | fit_intercept | TRUE |
| Logistic Regression | CV Round 1 | number_selected_features | 8 |
| Logistic Regression | CV Round 2 | penalty | "elasticnet" |
| Logistic Regression | CV Round 2 | C | 0,31 |
| Logistic Regression | CV Round 2 | l1_ratio | 0,7 |
| Logistic Regression | CV Round 2 | solver | "saga" |
| Logistic Regression | CV Round 2 | fit_intercept | TRUE |
| Logistic Regression | CV Round 2 | number_selected_features | 8 |
| Logistic Regression | CV Round 3 | penalty | "elasticnet" |
| Logistic Regression | CV Round 3 | C | 0,01 |
| Logistic Regression | CV Round 3 | l1_ratio | 0,9 |
| Logistic Regression | CV Round 3 | solver | "saga" |
| Logistic Regression | CV Round 3 | fit_intercept | FALSE |
| Logistic Regression | CV Round 3 | number_selected_features | 6 |
| Logistic Regression | CV Round 4 | penalty | "elasticnet" |
| Logistic Regression | CV Round 4 | C | 0,01 |
| Logistic Regression | CV Round 4 | l1_ratio | 0,9 |
| Logistic Regression | CV Round 4 | solver | "saga" |
| Logistic Regression | CV Round 4 | fit_intercept | TRUE |
| Logistic Regression | CV Round 4 | number_selected_features | 6 |
| Logistic Regression | CV Round 5 | penalty | "elasticnet" |
| Logistic Regression | CV Round 5 | C | 0,96 |
| Logistic Regression | CV Round 5 | l1_ratio | 0,3 |
| Logistic Regression | CV Round 5 | solver | "saga" |
| Logistic Regression | CV Round 5 | fit_intercept | TRUE |
| Logistic Regression | CV Round 5 | number_selected_features | 10 |
| Logistic Regression | CV Round 6 | penalty | "elasticnet" |
| Logistic Regression | CV Round 6 | C | 0,01 |
| Logistic Regression | CV Round 6 | l1_ratio | 0,9 |
| Logistic Regression | CV Round 6 | solver | "saga" |
| Logistic Regression | CV Round 6 | fit_intercept | TRUE |
| Logistic Regression | CV Round 6 | number_selected_features | 6 |
